# Topological measures for identifying and predicting the spread of complex contagions

**DOI:** 10.1038/s41467-021-24704-6

**Published:** 2021-07-20

**Authors:** Douglas Guilbeault, Damon Centola

**Affiliations:** 1 Haas School of Business, The University of California, Berkeley, Berkeley, CA USA; 2 The Annenberg School for Communication, The University of Pennsylvania, Philadelphia, PA USA; 3 School of Engineering, The University of Pennsylvania, Philadelphia, PA USA

**Keywords:** Society, Sociology

## Abstract

The standard measure of distance in social networks – average shortest path length – assumes a model of “simple” contagion, in which people only need exposure to influence from one peer to adopt the contagion. However, many social phenomena are “complex” contagions, for which people need exposure to multiple peers before they adopt. Here, we show that the classical measure of path length fails to define network connectedness and node centrality for complex contagions. Centrality measures and seeding strategies based on the classical definition of path length frequently misidentify the network features that are most effective for spreading complex contagions. To address these issues, we derive measures of *complex path length* and *complex centrality*, which significantly improve the capacity to identify the network structures and central individuals best suited for spreading complex contagions. We validate our theory using empirical data on the spread of a microfinance program in 43 rural Indian villages.

## Abstract (web summary)

Understanding of complex contagions is crucial for explaining diffusion processes in networks. Guilbeault and Centola introduce topological mechanisms and measures to elucidate spreading dynamics and identify the most influential nodes in social, epidemic and economic networks.

## Introduction

One of the most important network measures today is path length—defined as the shortest number of steps between any two vertices on a graph. This measure is considered to be a robust indicator of the typical distance between any two nodes in a network, such that the average shortest path length of a graph, also called its characteristic path length, is taken to identify a general topological property of all networks^1–5^. However, this measure of path length implicitly assumes a process of network traversal that relies on the theory of simple contagion, in which a single tie is sufficient for a contagion to travel from one node to another^1–5^. A key difficulty arises from the fact that many social contagions are “complex”, for which individuals require contact with multiple activated peers before they become activated themselves^6–9^. According to the standard measure of path length, network distance is the number of steps required to travel across a network, where each step is composed of a single tie^1–5^. Yet for complex contagions, such as the spread of new technologies^10,11^, health behaviors^8,9,11^, linguistic conventions^12,13^, internet memes^14^, social movements^15,16^, and political hashtags^17,18^, each step in the social network requires peer reinforcement from multiple ties. Measuring the path of a complex contagion thus requires measuring each step in the network not in terms of single ties, but rather in terms of reinforcing ties—typically referred to as wide bridges^6–9,11^.

Given the prevalence of complex contagion in social diffusion^6,11^, we argue that the classical measure of path length—hereafter “simple path length”—does not provide a satisfactory way to measure connectedness in social networks. Simple path length assumes that if a finite path of single ties exists between node *i* and node *j*, then a contagion can spread from node *i* to *j*^1–5^. However, numerous empirical and formal studies reveal the puzzling result that within social networks composed of a single connected component, it may nevertheless be impossible for a complex contagion to spread from one node to another^6–9,11^. The empirical frequency^10–18^ of studies which find that nodes are both topologically connected and yet socially disconnected for the transmission of social contagions indicates that simple path length does not provide a satisfactory measure of social distance and connectedness in social networks.

The inability for simple path length to properly measure network connectedness leads to a new challenge for longstanding solutions to the problem of identifying which individuals (i.e., seeds) in a social network are most influential for spreading a new behavior^19–26^. Well-established measures of node centrality (e.g., degree centrality^3^, betweenness centrality^27^, eigenvector centrality^3^, k-core centrality^3,28^, and percolation centality^24,25^) have become popular tools for characterizing the most influential nodes for the spread of social contagions in both theoretical and applied social networks^3,5,21–23,25,29^. Yet, several empirical studies of social diffusion have found that these measures of node centrality misidentify the most influential actors^15,22,30–33^. For example, recent findings on social media show the counterintuitive result that people with the highest betweenness and degree centrality are often not the most influential nodes for spreading political messages and controversial news, because these kinds of messages are complex contagions^6,17,32,33^.

Here, we show that the failure of popular measures of node centrality to detect node influence in the empirical spread of complex contagions is based on their consistent use of simple path length to calculate network connectedness. We find that measures of centrality that rely on simple path length are poorly adapted to predicting the diffusion (i.e., the peer-to-peer spread) of complex contagions. Specifically, all of the following measures of centrality rely on simple path length:

- Degree centrality: the centrality of a node is determined by the number of other nodes to which it is connected via single ties^3^.
- Betweenness centrality: the centrality of a node is determined by the number of shortest simple paths that pass through it^3^.
- Eigenvector centrality: the centrality of a node is determined by the number of single tie connections that a node shares with other nodes (specifically, accounting for each node’s single tie connections to other well-connected nodes)^3^.
- Optimal percolation centrality: the centrality of a node is determined by whether its removal collapses the largest connected component (which is defined in terms of simple paths) of a graph^24,25^. In practice, percolation centrality amounts to the product of the reduced degree centrality of a node and the total reduced degree centrality of all nodes at a given distance *d*, measured by simple path length.
- K-core centrality (also known as coreness^3,28^): the centrality of a node is determined by decomposing a network into subcomponents consisting of nodes connected with a degree of at least *k* or lower, where degree is measured in terms of single ties^3^.

**Fig. 1:**
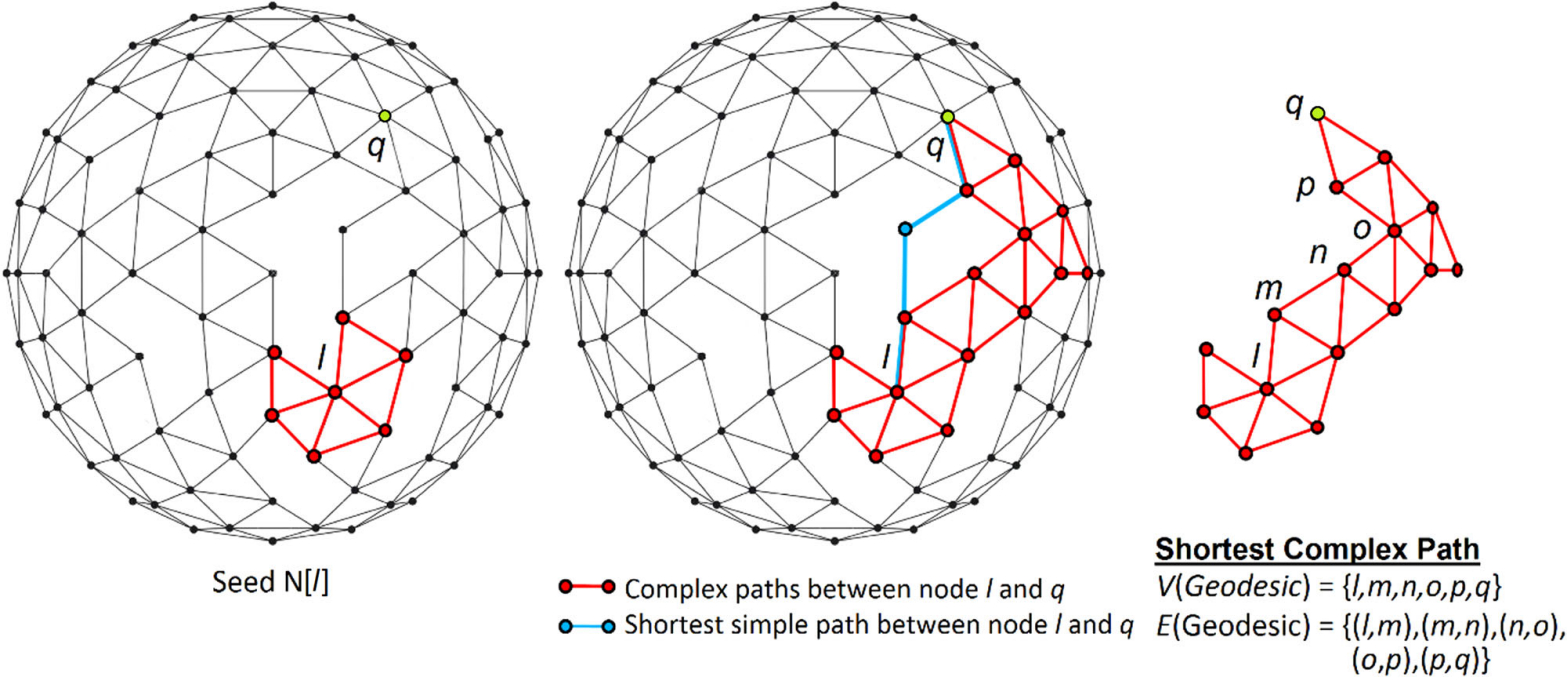
Identifying complex paths. This figure displays a visualization of the method for extracting the shortest complex path between nodes at any arbitrary distance in the network, as defined by Eqs. (1), (2), and (3) in the “Methods” section. The set of complex paths between seed neighborhood of node *l* and target node *q* (shown in green) is highlighted in red, while the shortest simple path length between *l* and *q* is highlighted in blue. This figure visualizes the complex path for a contagion where the adoption threshold is 2 for all nodes.

While recent studies have attempted to provide alternative definitions of centrality that overcome the limitations of standard approaches, these alternatives continue to rely on simple path length, typically selecting seeds with high levels of degree, betweenness, or eigenvector centrality^25,26,34–37^. None of these alternatives can explain the recurring empirical finding^6,11,15,21,30–33^ that nodes with high centrality (according to any of the measures that rely on simple path length) are ineffective for spreading complex social contagions. To address this problem, and the challenges it poses for contemporary conceptions of the relationship between network structure and social influence, we derive a measure of path length, called “complex path length” (PL_C_), which illuminates new, generalizable topological properties of connectedness and centrality (“complex centrality”), for all social networks. While our study is motivated by the theoretical challenges raised by empirical findings on the spread of complex contagions, we identify network measures that generalize across both simple and complex contagions, including all ranges of peer reinforcement that may be required for transmission (Fig. 1; see “Methods”).

We present our findings as follows: (1) First, in the interest of clarity, we define the general influence model for complex contagion. (2) Second, we derive a general topological measure for calculating the network distance between the nodes in a graph for the spread of a complex contagion: i.e., complex path length. (3) Third, we use the above results to derive a generalized measure of node centrality: i.e., complex centrality. (4) Fourth, we provide numerical results (with additional robustness tests in the Supplementary Material) demonstrating that complex path length provides an excellent match for estimating cascade frequency on simulated complex graphs. (5) Fifth, we provide numerical results demonstrating that complex centrality outperforms the most prominent measures of node centrality for identifying unique network locations most effective for “seeding” a complex social contagion. (These results are tested on a wide range of both artificial and empirical social networks, with additional robustness tests in the Supplementary Material.) (6) Sixth, we use the measures of complex path length and complex centrality to predict the most influential network locations for the spread of a microfinance program, as reported in an empirical study of social contagion in 43 Indian villages^38^.

For ease of presentation, in what follows we briefly describe our formal definitions and derivations, outlined in points 1–3 above. (The complete derivations are provided in the “Methods” section.) We then present findings 4–6 in detail, with supporting robustness tests (Supplementary Methods and Supplementary Notes 1–13).

To begin, we outline the formal influence model that underlies complex contagion. First, we define *G*(*V, E*) as an unweighted and undirected graph with a set of *n* agents, where *V* ≔ {1, …, *n*}, and a set of edges *E*. We denote the neighbors of node *i* as *N*[*i*]. In the complex contagion model, each node *i* ∈ *V* is assigned an adoption threshold *T*_i_ that specifies how many activated peers that node *i* has to be exposed to for *i* to adopt the contagion. Thresholds can be either absolute, where they specify the raw number of activated neighbors required to trigger adoption by node *i*, or they can be fractional, in which case they specify the fraction of *i*’s neighbors that must be activated to trigger adoption by *i*. Thresholds can be distributed homogeneously (i.e., held constant for all nodes) or heterogeneously, where each node receives a different threshold at some probability (e.g., where each node is assigned a threshold uniformly at random from a defined interval). Diffusion unfolds in discrete time steps: at step *t*, all nodes that were active in *t* − 1 stay active, and we activate any node *j* that has a sufficient number of activated neighbors to satisfy their threshold, *T*_j_.

We define the complex path between node *i* and node *j* as the sequence of neighborhoods through which a contagion must traverse to travel from the neighborhood of node *i*, *N*[*i*], to any node *j*, where *i, j* ∈ *V*. To characterize the structure and diffusion capacity of complex paths, we provide a new formal definition of bridge width that identifies whether the number of reinforcing ties between connected neighborhoods is sufficient to enable the spread of a complex contagion (see Supplementary Figs. 1 and 2, which visually demonstrate how bridge width is calculated on a comprehensive range of local neighborhood configurations). The complex path length between node *i* and node *j* is defined as the number of sufficiently wide bridges that are traversed as a complex contagion spreads from *N*[*i*] to node *j* (Eqs. (1–3) in “Methods”). We use this method to identify chains of bridges between nodes at any distance in the network, and for characterizing the length and width of these chains (Fig. 1). This definition of network connectedness, PL_C_, motivates a new measure of node centrality for complex contagions (Eqs. (5–7) in “Methods”), i.e., complex centrality (CC). The complex centrality of a node *i* (CC_i_) is the average length of the complex paths extending from the neighborhood of node *i*, *N*[*i*], denoted by ${{{\mathrm{PL}}}}_{{{{\mathrm{C}}}}_{\mathrm {i}}}$ (Eq. (5) in “Methods”). The node with the highest complex centrality in a graph is the node with the highest average complex path length, max [${{{\mathrm{PL}}}}_{{{{\mathrm{C}}}}_{\mathrm {i}}}$]^*N*^_*i*=1_ (Eq. (7) in “Methods”).

## Results

We begin by showing how our measure of bridge width effectively captures the connectedness of a social network for the spread of complex contagion. To present our findings in a way that is consistent with canonical work on connectedness and path length^1,2^, we begin by studying a continuum of k-regular graphs generated using the same approach adopted by this canonical work^2,7^. Specifically, we start with a ring of *n* vertices in a regular lattice, each connected to *k* nearest neighbors, and we generate a continuum of k-regular graphs by rewiring pairs of edges chosen uniformly at random around the ring (with probability *p*), ensuring that all rewired edges are degree-preserving and maintain the k-regular degree distribution^7,8^. Previous work^1,2^ found that increasing the randomness of a graph substantially decreases the average shortest simple path length between all nodes, which accelerates the spread of simple contagions^1,2^. However, we find the opposite effect for the spread of complex contagions, for which increasing randomness in network structure disrupts the fraction of sufficiently wide bridges, leading to sharp declines in the capacity for complex contagions to spread. Here, we analyze every neighborhood bridge in each graph and calculate the proportion of bridges that are sufficiently wide to enable diffusion. We refer to this quantity as the proportion of locally sufficient bridges (LB) (“Methods”). We use LB to estimate the capacity of a graph to support global cascades of complex contagions, which we calculate by averaging the diffusion outcomes that result from attempting to use each node and its neighborhood as the initial seeds for a diffusion process. After demonstrating these results for randomization on regular graphs, we then generalize these findings for random scale-free graphs (the full set of robustness tests are included in the *Supplementary Material*).

**Fig. 2:**
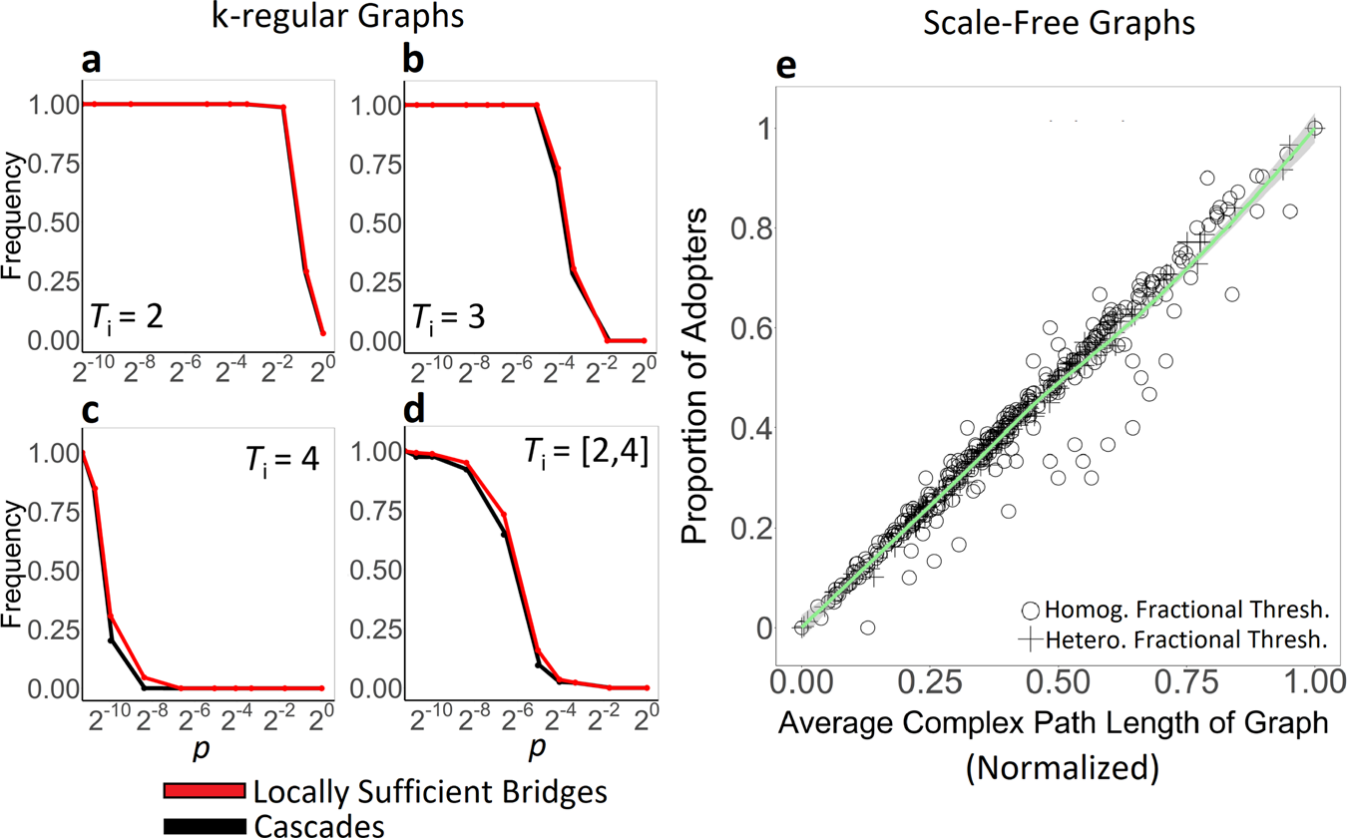
Using bridge width and complex path length to estimate the frequency of global cascades. We assume a constrained seeding budget, such that we evaluate cascade frequency for the minimum number of seed nodes sufficient to trigger a given threshold, *T*. For each threshold value, *T*, we conducted 1000 independent simulation trials on each graph, each of which began by initially activating a focal node and a random subset of its neighborhood, corresponding to *T*_i_ − 1 (i.e., the threshold of *T*_i_ as indicated by the panel minus 1 for node *i*). This procedure exhaustively explored all possible focal nodes in each network (without replacement). For each graph configuration (e.g., value of *p*), and for each value of *T*, we replicated the full ensemble of 1000 simulation trials across 50 distinct network realizations. Results show the average cascade frequency across all replications. The fit between the frequency of locally sufficient bridges and the frequency of global cascades on k-regular graphs (*N* = 1000, 〈k〉 = 8, replicated over all possible seed nodes), shown for **a** *T*_i_ = 2; **b** *T*_i_ = 3; **c** *T*_i_ = 4; and **d** heterogeneously distributed (*T*_i_ = [2, 4]). **e** The fit between the average complex path length of a graph and the proportion of adopters in scale-free graphs with homogeneous fractional thresholds (*N* = 1000; *γ* = 3; *m* = 4; *p* = 0.5; *T*_i_ = 0.1, *T*_i_ = 0.2, *T*_i_ = 0.3, *T*_i_ = 0.4, *T*_i_ = 0.5) and heterogeneous fractional thresholds (*N* = 1000; *γ* = 3; *m* = 4; *p* = 0.5; *T*_i_ = [0.1, 0.5]). Homog., homogeneous. Hetero., heterogeneous. Thresh., thresholds.

Panels a–d of Fig. 2 show that as graphs become increasingly random, the frequency of global cascades decreases precipitously along with the average proportion of sufficiently wide bridges. The frequency of locally sufficient bridges accurately estimates the capacity for k-regular graphs with varying levels of randomness to support global cascades of complex contagions—regardless of whether thresholds are homogeneously or heterogeneously distributed. Put another way, the frequency of locally sufficient bridges in a graph provides an effective measure of a graph’s connectedness—that is, the graph’s ability to support the spread of a complex contagion between any two nodes in the population.

Panel e of Fig. 2 generalizes these results to random scale-free networks by examining average complex path length. Since complex paths are formed by chains of sufficiently wide bridges, it follows that the average complex path length of a graph should effectively estimate the fraction of nodes in a population that can be reached by a complex contagion. Panel e shows that the average complex path length of a complex (scale-free) graph accurately estimates the average size of the cascades generated across all possible seed neighborhoods in the network, using both homogenous fractional thresholds (*T*_i_ = 0.1, *T*_i_ = 0.2, *T*_i_ = 0.3, *T*_i_ = 0.4, *T*_i_ = 0.5; *p* < 0.0001, *r*_*s*_ = 0.95, CI = [0.92, 0.97]) and heterogeneous fractional thresholds (*T*_i_ = [0.1, 0.5]; *p* < 0.0001, *r*_*s*_ = 0.99, CI = [0.99, 1.0]). These findings are robust for homogeneous and heterogeneous distributions of absolute thresholds (Supplementary Figs. 7 and 8), and for k-regular graphs (Supplementary Fig. 9).

The above findings on complex path length offer a new approach, called complex centrality, for addressing the longstanding problem of identifying the most influential (i.e., the most central) nodes in a network diffusion process. In what follows, we evaluate the performance of competing measures of centrality for identifying the most influential seed nodes for spreading social contagions within a set of empirical social networks collected for studying the spread of public health behaviors^39^ (e.g., wearing face masks during the COVID-19 pandemic). We use 74 empirical social networks taken from the Add Health dataset—the largest publicly available collection of adolescent social networks drawn from over 70 distinct communities in the US^39^ (see “Methods” for further detail on this dataset). In each network, we compare the effectiveness of nodes with the highest complex centrality against the nodes identified by each of the theoretically defined measures of the most “central” network locations—i.e., by degree centrality, betweenness centrality, eigenvector centrality, k-core centrality, and percolation centrality—and evaluate the spread of complex social contagions of varying thresholds.

We initiated each test of each centrality measure by activating the theoretically identified seed node (according to each theory of node centrality) and a random subset of its neighborhood sufficient to trigger subsequent adoption (i.e., the current threshold of *T*_i_ minus 1 for node *i*) (Supplementary Fig. 4). For instance, for *T* = 3 we would initiate diffusion by activating the identified seed node and 2 of its randomly selected neighbors (see “Methods” for further details on our approach).

**Fig. 3:**
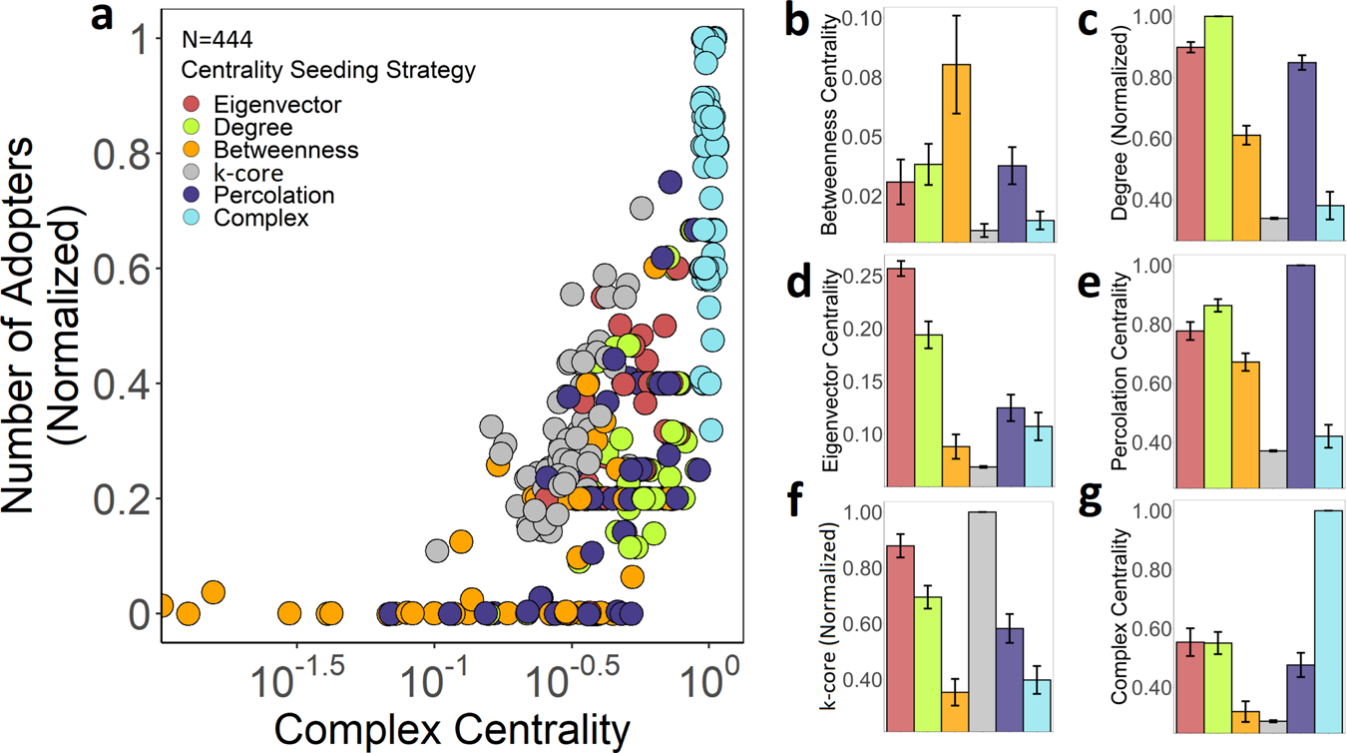
Comparing seeding strategies by simulating diffusion in empirical social networks. Diffusion results are displayed for seeding strategies based on node centrality for 74 Add Health networks, across a range of homogeneously distributed absolute thresholds for complex contagion (*T*_i_ = 2, *T*_i_ = 3, *T*_i_ = 4, *T*_i_ = 5, and *T*_i_ = 6). For each network under each *T* regime, we identify the most central focal node for each seeding strategy and simulate diffusion by activating this focal node and *T*_i_ –1 of its neighbors (i.e., the current threshold of *T*_i_  minus 1 for node *i*). **a** Each datapoint represents the success of diffusion and complex centrality (averaged across all threshold values) for each seeding strategy on each Add Health network. Six seeding strategies across 74 networks yield a total of 444 datapoints. To average the diffusion outcomes on the same graph across different homogeneous threshold conditions, the final number of adopters and complex centrality were standardized using min–max normalization for each threshold condition prior to averaging. **a** For each datapoint, the vertical axis represents the success of diffusion, and the horizontal axis represents the measure of complex centrality for the focal nodes used for each seeding strategy (regardless of the centrality measure that selected that node). Results are averaged across all threshold values on each Add Health network. Slight horizontal jittering is used to reveal overlapping points (*δ* = 0.01). This normalization technique captures the average ranking of each seeding strategy on each network, averaged across threshold regimes. Additional panels show comparisons across all seeds according to their **b** betweenness centrality, **c** degree centrality (normalized), **d** eigenvector centrality, **e** percolation centrality (normalized; *d* = 3), **f** K-core centrality (normalized), and **g** complex centrality. Error bars show 95% confidence intervals.

Based on prior studies of complex contagion^6,7^ and for clarity of exposition, we present our basic findings for populations with homogeneously distributed absolute thresholds ranging from *T*_i_ = 2 to *T*_i_ = 6. (For *T*_i_ = 1, all strategies produced complete global adoption; for *T*_i_ > 6, we observed minimal spreading across all networks.) Our complete results (provided in the *SI*) show these findings to be robust to both homogeneous and heterogeneous threshold distributions, and to the use of absolute or fractional thresholds. In the results presented below, each network produced 6 observations (one for each seeding strategy) for each of the 5 values of *T*_i_ (*T*_i_ = 2, *T*_i_ = 3, *T*_i_ = 4, *T*_i_ = 5, *T*_i_ = 6). This produced 30 observations for each network, and 2220 observations in total. To provide a summary result for each strategy, we averaged the diffusion success over all thresholds for each seeding strategy on each network. This produced 444 observations in total, as shown in Fig. 3.

Panel a of Fig. 3 shows that seeds with the highest complex centrality generated significantly more adopters than seeds with the highest degree centrality (*n* = 148, *p* < 0.001, CI = [0.41, 0.51]), betweenness centrality (*n* = 148, *p* < 0.001, CI = [0.55, 0.66]), eigenvector centrality (*n* = 148, *p* < 0.001, CI = [0.35, 0.50]), k-core centrality (*n* = 148, *p* < 0.001, CI = [0.38, 0.49]), and percolation centrality (*n* = 148, *p* < 0.001, CI = [0.46, 0.59]), across all Add Health networks (Wilcoxon signed-rank test, two-tailed). The horizontal axis in panel a indicates that the complex centrality of a node effectively identifies its overall influence in the network, regardless of the particular seeding strategy that selected that node (*n* = 444, *p* < 0.001, *r* *=* 0.77, CI = [0.72, 0.81], two-tailed). (These results are robust to comparing seeding strategies within each threshold regime separately; Supplementary Fig. 10, Supplementary Table 1). Figure 3b–f shows that complex centrality identifies influential nodes with qualitatively distinct topological positions as compared to the nodes identified by centrality measures based on simple path length (Supplementary Note 12). Perhaps most surprisingly, complex centrality identifies seed nodes that have low influence according to the most popular centrality measures.

Our supplementary analyses (Supplementary Notes 1–13) show that these results are robust across a wide range of theoretical network conditions. In brief, we use a diverse ensemble of simulated scale-free networks to test a variety of common social influence models, including: (1) the complex contagion model, using heterogeneous distributions of absolute thresholds; (2) the complex contagion model, using heterogeneous distributions of fractional thresholds; (3) the Independent Cascade model^26^; and (4) the Linear Threshold^26^ model (Supplementary Fig. 11). We show that our results also hold when (i) varying the amount of clustering in scale-free networks using Holme and Kim’s tuning algorithm^40^ (Supplementary Fig. 12), (ii) using homogeneous distributions of absolute and fractional thresholds in the complex contagion model (Supplementary Fig. 13), (iii) considering different values of *θ* (the activation parameter) in the Independent Cascade model (Supplementary Fig. 14), (iv) comparing complex centrality against additional centrality measures based on simple path length that are not typically used for seeding, such as closeness centrality and reach centrality (Supplementary Fig. 15), (v) holding node degree constant in k-regular networks (Supplementary Fig. 16), (vi) with conventionally generated scale-free networks^3^ (Supplementary Fig. 17), and (vii) when varying the parameter *d* for measuring optimal distance in percolation centrality^24,25^ (Supplementary Fig. 18). In addition, our supplementary analyses show that, across various topologies and influence models, complex centrality also identifies influential seeds for the spread of complex contagions more effectively than a canonical greedy algorithm, which simulates diffusion from every possible seed and selects the set of seeds with the greatest expected diffusion based on their individual performance (Supplementary Fig. 11)^26^.

To test the effectiveness of our network measures of complex path length and complex centrality for predicting the effects of network structure on an empirical diffusion process, we evaluate the theoretical predictions of complex path length and complex centrality using an empirical network study on the spread of a microfinance program in rural India^38^. These data offer an unusually comprehensive record of network diffusion, collected from 43 distinct villages in which complete network data were recorded for every village, along with a complete record of the spreading dynamics of a novel microfinance program (referred to as the Bharatha Swamukti Samsthe, or BSS, program) across every household in every village (see “Methods” for details on the data for each of the village networks and their associated adoption dynamics)^38,41^. These data offer an exceptionally robust test of our hypotheses that (i) complex path length will be predictive of the village networks in which the microfinance program will spread most effectively, and (ii) complex centrality will be predictive of the specific households in each village that will be most influential in spreading the BSS program.

**Fig. 4:**
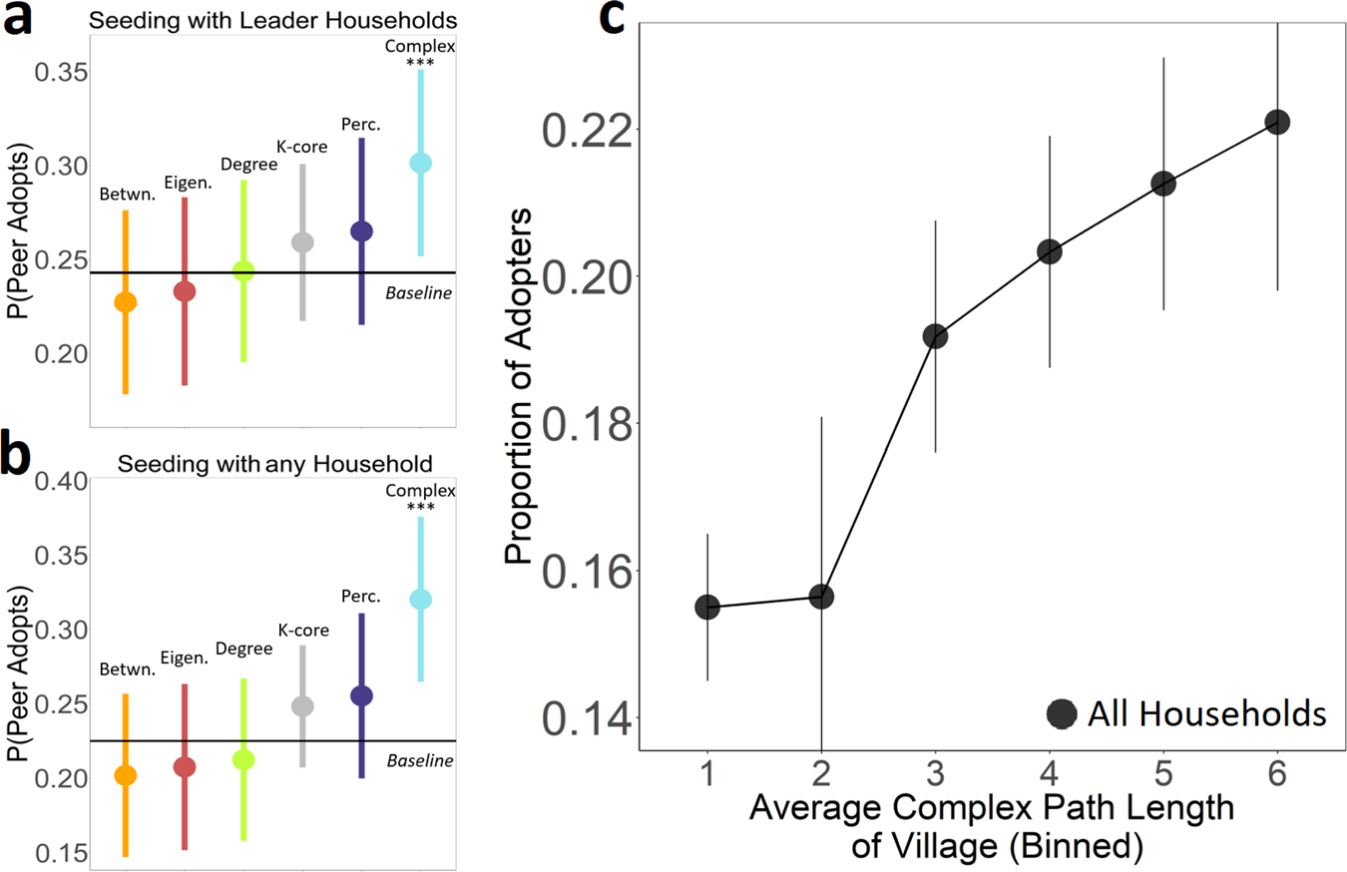
Using node centrality to predict the empirical diffusion of the Bharatha Swamukti Samsthe (BSS) microfinance program in rural India. Empirical diffusion results are displayed for seeding strategies based on node centrality for 43 rural Indian villages. Bars represent the probability that the network neighbors of a seed household adopt the contagion once the household has adopted, averaged across all 43 rural villages. **a** Displays these results when selecting seeds only from the set of village leaders who were empirically found to initially adopt the BSS program and agree to assist in its diffusion (referred to as ‘leader’ households); and **b** displays these results selecting seeds from any possible household in the village. Forty-three network observations across 6 distinct centrality measures yield 258 observations for each panel. Panel **c** displays the relationship between the average complex path length of a village and the overall fraction of the village that consequently adopted the BSS microfinance program. For percolation centrality, the optimal distance *d* is set to 3. Error bars display a standard deviation. Betwn., betweenness centrality; Eigen., eigenvector centrality; Perc., percolation centrality. Baseline indicates the expected diffusion outcomes when randomly selecting seed households from the possible seeds identified by the competing centrality measures, not including complex centrality.

We calculated each village’s average complex path length and each household’s average complex centrality within each village using an ensemble of estimated threshold distributions for each village (see “Methods” for details on this estimation procedure; see Supplementary Fig. 6 for a schematic of this analytic approach). Similar to the approach adopted in Fig. 3, we evaluated the correlation between the complex centrality of each household in every village and the resulting spread of the microfinance program from each adopting household to its network contacts. Figure 4 panel a shows the results for “leader” households (i.e., households specifically selected during the original study to initiate the microfinance program within each village), and panel b shows the results for “regular” households (i.e., all households from all 43 villages, many of which were subsequent adopters of the program who helped to spread it through their respective villages). To provide a reference point for our results, panels a and b in Fig. 4 also report the same analyses for all of the centrality measures discussed above: degree, eigenvector, betweenness, k-core, and percolation (see Supplementary Tables 2 and 3 for complete details). In addition, similar to the approach adopted in Fig. 2 above, we also evaluated the correlation between each village’s complex path length and the overall rate of adoption in each village (as shown in Fig. 4 panel c, below).

Panel a of Fig. 4 shows that leader households with the highest complex centrality are associated with a significantly higher probability of inducing the spread of the microfinance program than the leader households that had the highest centrality scores based on existing measures of node centrality (*n* = 258, *p* = 0.003, CI = [0.24, 0.33]; see Supplementary Table 2 for details on statistical analysis and controls). Notably, complex centrality is the only topological measure that was able to identify the leader households that had a significant impact on increasing the spread of the microfinance program (relative to the baseline expectations for the program generated by selecting at random leader seed households identified by competing centrality measures, see Supplementary Methods). Panel b of Fig. 4 replicates the same results while broadening the search for seeds beyond the pre-specified leaders selected by Banerjee et al.^38^ Across every possible household in each village, households with the highest complex centrality are associated with a significantly higher probability of inducing the adoption of the microfinance program among their network neighbors (*n* = 258, *p* < 0.001, CI = [0.26, 0.37]; see Table S2 for details on statistical analysis). Supplementary analyses show that these results are robust to (i) controlling for the full range of socioeconomic variables associated with each household in the Banerjee et al. dataset^38,41^ (Supplementary Tables 2 and 3), (ii) clustering standard errors at the village-level to adjust for non-independence, (iii) when examining adoption solely among non-leader households, and (iv) when exclusively examining the ability of non-leader households to trigger adoption of the BSS program (Supplementary Note 14).

Panel c of Fig. 4 tests the village-level hypothesis that the average complex path length of an entire village is positively correlated with the overall rate of adoption of the microfinance program. Consistent with the theoretical predictions displayed in Fig. 2, panel c of Fig. 4 shows that villages with higher average complex path length exhibited significantly greater program adoption (*p* = 0.009, *r*_*s*_ = 0.41, *n* = 43, CI = [0.04, 0.69], two-tailed). By contrast, the average simple path length of each village fails to predict the overall capacity of the village to spread the microfinance program (*p* = 0.1, *r*_*s*_ = −0.27, *n* = 43, CI = [−0.58, 0.07], two-tailed).

## Discussion

Path length is one of the most important and influential measures of network structure. It underlies nearly every theory of social connectedness, social distance, and social influence within social networks. Here we show that the classical measure of simple path length, upon which most popular measures of node centrality depend, implicitly assumes the spreading dynamics of simple contagion. This assumption has resulted in several puzzling empirical findings in which individuals with putatively low centrality have been shown to be more influential for diffusion than individuals with high centrality (according to prominent measures of degree centrality, betweenness centrality, eigenvector centrality, k-core centrality, and percolation centrality). We derive new topological definitions of bridge width, path length, and centrality, which provide general topological measures for accurately estimating the network properties of connectedness, distance, and centrality for the spread of complex social contagions. We find that these measures offer significant theoretical improvements over existing measures of population-level network topology, and individual-level node centrality, for predicting the network properties that will increase the spread of complex social contagions.

Our findings offer several noteworthy departures from the dominant strategies for applying network theory to problems of social diffusion^1,3,5,29,42–47^. First, a common assumption among both theoretical and applied studies of network diffusion is that people with more connections are more influential^5,21,22,29,30,42–45^. Our findings disagree with the frequently asserted claim in this literature that degree centrality is an effective, if approximate, means of identifying the most influential individuals within a social network, regardless of context^5,21,22,29,30,42–45^. Second, a common assumption within organizational studies of social networks is that information brokers—i.e., people who participate in multiple distinct network communities that are largely disconnected—have outsized influence because they are the gatekeepers in the flow of contagions between communities^46,47^. This assumption has resulted in betweenness centrality becoming one of the most widely used measures of network influence within organizational theory^1,27,29,42,43,46–49^. By contrast, our findings indicate that network locations with low degree centrality and low betweenness centrality may nevertheless be the most influential locations in the population. We also find that individuals with the highest levels of degree centrality and betweenness centrality typically occupy ineffective network positions for initiating the spread of complex social contagions—including health behaviors^8,9^, linguistic conventions^6,12,13^, political memes^14^, social movements^15,16^, and complementary technologies^6,10^. We anticipate that an important direction for future work will be the exploration of new algorithms for computing the theoretical properties of complex path length and complex centrality, which may benefit from recent developments that improve the scalability of novel algorithmic techniques^50^. Another interesting direction for future research is the application of our topological measures for identifying specific network locations that can be used to efficiently stop the spread of an existing complex contagion from one part of a network to the entire population (akin to the problem of network “immunization” for simple contagions)^6,51,52^.

## Methods

Here we provide a formal logic for representing and reproducing our measures of bridge width, complex paths, and complex centrality. Supplementary Figs. 1 and 2 provide a step-by-step guide for how to identify the network structures captured by our measures, as well as how to calculate bridge width across a comprehensive range of neighborhood configurations.

### Definition of local measure of sufficient bridge width

To describe complex paths, we provide a formal definition of bridge width that provides (i) a method for measuring the local connectivity of bridges across all nodes, (ii) a method for identifying chains of bridges between nodes at any distance in the network, and (iii) a method for measuring the length of these chains. We begin by considering an unweighted undirected graph *G* with the set of vertices *V* and edge set *E*. We define the complex path between node *i* and node *j* as the sequence of neighborhoods through which a complex contagion must traverse to travel from the neighborhood of *i*, *N*[*i*], to any node *j*, where *i, j* ∈ *V*. We measure the connectivity between two neighborhoods using the concept of a bridge between neighborhoods (defined below). If a bridge between two neighborhoods is “wide” enough to support diffusion, it is called a “sufficient bridge”. For a contagion with an adoption threshold *T*_j_ (defined for each node *j*), a sufficient bridge exists from node *i*’s neighborhood to node *j*’s neighborhood if and only if the following conditions are met. For simplicity, we specify these conditions assuming that *T*_j_ is homogeneous across all nodes, but these measures can be readily adapted to heterogeneous distributions of *T*_j_ (discussed below).

Let *N*[*i*] refer to the closed neighborhood of node *i*, defined as the induced subgraph of *G* including all vertices adjacent to *i*, along with *i*.

Let E(*N*[*i*]) indicate the edge set of the neighborhood of node *i*, including all ties to *i* within *N*[*i*].

Let *T*_j_ refer to the adoption threshold of node *j* (i.e., the number or fraction of activated peers that node *j* needs to encounter to adopt).

Let O_ij_ refer to the overlap (intersection) between *N*[*i*] and *N*[*j*], i.e., O_ij_ ≡ *N*[*i*] ∩ *N*[*j*].

Let D_ij_ refer to the disjoint set of nodes in *N*[*j*] that are not in *N*[*i*], such that ∀*v*(*v* ∈ D_ij_ → *v* ∈ *N*[*j*] ∧ *v* ∉ *N*[*i*]).

Let R_ij_ refer to the “reinforcement” set of nodes, which consists of the nodes in D_ij_ that are connected to the nodes in *N*[*i*]. Formally, ∀*v*(*v* ∈ R_ij_ → *v* ∈ D_ij_ ∧ |E(*N*[*i*]) ∩ E(*N*[*v*])| ≥ 1).

Let the bridge between node *i* and *j* be defined as the union of O_ij_ and R_ij_, i.e., BW_ij_ ≡ O_ij_ ⋃ R_ij_.

Let the width of the bridge between *i* and *j* be defined as W_ij_, where W_ij_ ≡ |BW_ij_| (the cardinality of the bridge between node *i* and *j*).

Under the above definitions, the bridge between *N*[*i*] and *N*[*j*] can support the spread of a contagion—i.e., the bridge is locally sufficient—if W_ij_ ≥ *T*_j_.

We ascribe every bridge in *G* a binary value indicating whether the bridge is sufficiently wide to enable diffusion. We indicate this binary value in notation by placing sharp brackets around the term for bridge width:

1 $$[{{{{\rm{W}}}}}_{{{{\rm{ij}}}}}]\equiv \left\{\begin{array}{c}1\;{{{\rm{if}}}}\,{{{{\rm{W}}}}}_{{{{\rm{ij}}}}}\ge {T}_{{{{\rm{j}}}}}\\ 0\;{{{\rm{otherwise}}}}\end{array}\right.$$

The above definition of bridge width can be readily adapted to heterogeneous distributions of thresholds by requiring that R_ij_ consists only of nodes from D_ij_ that can be activated by *N*[*i*]. Specifically, this requires that we keep each node *x* in R_ij_ only if O_ix_ ≥ *T*_x_—i.e., if there are enough ties from *N*[*i*] to satisfy *T*_x_.

This quantity provides a robust measure for the local connectivity of a network, defined via the following procedure:

Let B_i_ refer to the subset of nodes from *V* that locally share a bridge with *N*[*i*]. Formally, ∀*v*(*v* ∈ B_i_ → *v* ∈ *V* ∧ *v* ≠ *i* ∧ W_vi_ ≥ 1).

We can use these measures to calculate the proportion of local bridges that are sufficiently wide for a node to spread a contagion beyond its neighborhood:

2 $${{{\rm{LB}}}_{{{{\rm{i}}}}}}\equiv \frac{1}{|{{{\rm{V}}}}({{{{\rm{B}}}}}_{{{{\rm{i}}}}})|}\cdot \sum \forall x(x\in {{{{\rm{B}}}}}_{{{{\rm{i}}}}}\to [{{{{\rm{W}}}}}_{{{{\rm{ix}}}}}])$$

This local measure can then be averaged across all nodes in a graph:

3 $${{{\rm{LB}}}}\equiv \frac{1}{n}\mathop{\sum }\limits_{i=1}^{n}{{{\rm{L{B}}}}}_{{{{\rm{i}}}}}$$

### Definition of complex path length

The complex path length (PL_c_) between nodes *i* and *j* is defined as the number of sufficient bridges that are traversed in the complex path between *N*[*i*] and node *j* (Fig. 1). We assume that if a contagion cannot spread from *N*[*i*] to node *j*, then ${{{\mathrm{PL}}}}_{{{{\mathrm{C}}}}_{\mathrm {ij}}}$ = 0. As visualized in Fig. 1, we define complex paths and their length through the following procedure:

Let CP_ij_ refer to the induced subgraph of nodes activated when spreading a complex contagion from *N*[*i*] to node *j*, which contains the set of possible complex paths between *N*[*i*] and node *j*_._

Let ${{{\mathrm{GEO}}}}_{{{{\mathrm{CP}}}}_{\mathrm {ij}}}$ refer to the geodesic between node *i* and node *j* within CP_ij_, which identifies the shortest complex path within CP_ij_.

Let *ϕ*(${{{\mathrm{GEO}}}}_{{{{\mathrm{CP}}}}_{\mathrm {ij}}}$) refer to the vertex sequence in GEOCP_ij_.

The complex path length (${{{\mathrm{PL}}}}_{{{{\mathrm{C}}}}_{\mathrm {ij}}}$) between *N*[*i*] and node *j* is thus defined as:

4 $${{{{\rm{PL}}}}}_{{{{{\rm{c}}}}}_{{{{\rm{ij}}}}}}\equiv |\phi ({{{{\rm{GEO}}}}}_{{{{{\rm{CP}}}}}_{{{{\rm{ij}}}}}})|$$

The average ${{{\mathrm{PL}}}}_{{{{\mathrm{C}}}}_{\mathrm {i}}}$ (for a given node *i*) is given by:

5 $${{{{\rm{PL}}}}}_{{{{{\rm{c}}}}}_{{{{\rm{i}}}}}}\equiv \frac{1}{n-|{{{\rm{V}}}}(N[i])|}\cdot \mathop{\sum}\limits_{i\ne {{{\rm{j}}}}}{{{{\rm{PL}}}}}_{{{{{\rm{c}}}}}_{{{{\rm{ij}}}}}}$$

Finally, the global complex path length of *G* is determined by averaging across the average complex path length of all nodes in *G*, giving:

6 $${{{{\rm{PL}}}}}_{{{{\rm{c}}}}}\equiv \frac{1}{n}\mathop{\sum }\limits_{i=1}^{n}{{{{\rm{PL}}}}}_{{{{{\rm{c}}}}}_{{{{\rm{i}}}}}}$$

### Definition of complex centrality

The definition of complex path length yields a new measure, called complex centrality (CC). Similar to the definition of degree centrality^3^, the complex centrality of a node *i* (CC_i_) is ${{{\mathrm{PL}}}}_{{{{\mathrm{C}}}}_{\mathrm {i}}}$, where the node with the highest complex centrality in a graph is the node with the highest average complex path length, formally expressed as:

7 $${{{\rm{max}}}}[{{{{\rm{PL}}}}}_{{{{{\rm{c}}}}}_{{{{\rm{i}}}}}}]{\,}_{i=1}^{N}$$

By defining a node’s centrality in terms of its highest average complex path length, this method identifies the nodes in any graph, and for any contagion, that have the longest and most robust chains of sufficient bridges that reach the greatest number of target nodes.

### Description of the Add Health network dataset

The Add Health dataset was constructed from an in-school survey, administered to 90,118 students from over 70 distinct communities throughout the US in 1994–1995^39^. All network data is available at this github: https://github.com/drguilbe/complexpaths^53^. The Add Health survey was designed to gather data on students’ social networks. Each student was given a paper-and-pencil questionnaire and a copy of a roster listing every student in the school and, if the community had two schools, the students were provided with the roster of the “sister” school. Students were asked to “List your closest (male/female) friends. List your best (male/female) friend first, then your next best friend, and so on. (Girls/boys) may include (boys/girls) who are friends and (boy/girl) friends”. This dataset was chosen for the purposes of our study because the social networks possess high levels of topological variation, in terms of population size, average degree (all with nonuniform degree distributions), and average clustering (Supplementary Fig. 3).

### Simulating diffusion on the Add Health dataset

For this analysis, we initiate diffusion by initially activating each possible seed and a random subset of its neighborhood corresponding to *T*_i_ − 1 (i.e., the current threshold of *T*_i_ minus 1 for node *i*) (Supplementary Fig. 4). For example, when *T*_i_ = 3, we initiate diffusion from every possible seed node by first activating that node and 2 of its randomly selected neighbors, and then we observe the simulated contagion process. Given the importance of clustered social influence for complex contagions, we adopt a clustered seeding strategy, such that if the seeding budget exceeds the size of the most central node’s neighborhood, we iteratively activate nodes that are directly connected to the neighbors of the most central node until we reach the corresponding seeding budget. Once diffusion has been attempted from every possible seed node, we then compare the ability for each centrality measure to accurately identify the most influential seeds. Consistent with the canonical complex contagion model^6,7^, we studied populations with homogeneously distributed absolute thresholds ranging from *T*_i_ = 2 to *T*_i_ = 6. (For *T*_i_ = 1, all strategies produced complete global adoption; for *T*_i_ > 6, we observed minimal spreading across all networks.) All results are robust to using homogeneous or heterogeneous distributions of either absolute or fractional thresholds (Supplementary Notes 1–11). Each network produced 6 observations (one for each seeding strategy) for each of the 5 values of *T*_i_ (*T*_i_ = 2, *T*_i_ = 3, *T*_i_ = 4, *T*_i_ = 5, *T*_i_ = 6).

### Description of the Banerjee et al. dataset on microfinance diffusion in rural India

The empirical dataset examined for Fig. 4 derives from Banerjee et al.^38,41^, who collected information about social networks and tracked the adopters of a microfinance program (referred to as the Bharatha Swamukti Samsthe, BSS, program) among all households in 43 distinct villages. In each of the 43 villages, the microfinance program was first introduced to the town leaders, who were asked to organize a meeting at which their followers could be informed about the microfinance program and its benefits. Crucially, Banerjee et al. monitored whether each household in each village adopted the microfinance program overtime, with the ability to link their adoption of the BSS program to each household’s position in the village’s social network, both with respect to the leaders who seeded the program, and also with respect to the households without leaders that adopted and provided reinforcement for other households to follow suit. This data thus supports an analysis of how the BSS program spread as a social contagion.

To measure the social network structure of each village, Banerjee et al. administered surveys to each household, which identified social relations across twelve dimensions: those who visit the respondent’s home, those whose homes the respondent visits, kin in the village, nonrelatives with whom the respondent socializes, those from whom the respondent receives medical advice, those from whom the respondent would borrow money, those to whom the respondent would lend money, those from whom the respondent would borrow material goods (e.g., kerosene and rice), those to whom the respondent would lend material goods, those from whom the respondent gets advice, those to whom the respondent gives advice, and those with whom the respondent goes to pray (at a temple, church, or mosque). Banerjee et al. showed how all of these measures can be combined to form a single binary, bidirectional network, where two households are represented as being connected by a single tie if they are connected through at least one of the twelve social dimensions above. A unique strength of this dataset is that Banerjee et al.’s survey also associated each household with a range of demographic and socioeconomic variables—such as the number of beds in the household and whether it has electricity—which can be used as statistical controls when estimating the effect of node centrality on the ability for households to trigger adoption of the BSS program among their network peers. Supplementary Fig. 5 indicates that these villages possessed significant topological variation, in terms of population size, average degree (all with nonuniform degree distributions), and average clustering.

### Calculating the average complex centrality of nodes in Banerjee et al.’s dataset on microfinance diffusion in rural India

Since it is not possible to directly determine the empirical adoption thresholds that characterized each household’s willingness to adopt, we calculated a household’s expected complex centrality as its average centrality across a range of adoption thresholds (see Supplementary Fig. 6 for a schematic of this analytic approach). We first simulate diffusion from each household while holding the thresholds of all households constant across a range of absolute adoption thresholds, from *T*_i_ = 2 to *T*_i_ = 6. For example, we set the adoption threshold of each household to *T*_i_ = 2 and then we simulate diffusion when seeding from each possible household. Similar to our Add Health simulation, we adopt a clustered seeding approach. We take the same approach for each *T*_i_ from *T*_i_ = 2 to *T*_i_ = 6. In each case, when activating a given household as the seed, we set the number of nodes to activate from the seed’s neighborhood to *T*_i_ − 1. We then take the average of each household’s complex centrality across each value of *T*_i_. As the final step, for each village, we identify the household with the highest centrality according to each centrality measure—degree, eigenvector, betweenness, k-core, and percolation—in addition to identifying the node with the highest average complex centrality. To evaluate our predictions, we compare the ability for each centrality measure to identify influential households, where an empirical measure of household influence is determined by measuring the fraction of a household’s neighbors who adopted after the seed household adopted (see Supplementary Tables 2 and 3 for full details on our statistical approach).

## Supplementary information

Supplementary Information

## Data Availability

The data in this study have been deposited on github and are available here: https://github.com/drguilbe/complexpaths^53^. Source data are provided with this paper.

## Source data

Source Data

## Code availability

Code for replicating this study has been deposited on github and is available here: https://github.com/drguilbe/complexpaths^53^.
