# Supplementary material for: Topological measures for identifying and predicting the spread of complex contagions
Source: Nat Commun. 2021 Jul 20;12:4430. doi: 10.1038/s41467-021-24704-6 (PMC8292353; doi:10.1038/s41467-021-24704-6)
Supplement: Supplementary file 1 — Supplementary Information [file 41467_2021_24704_MOESM1_ESM.pdf]

Supplementary Information for

Topological Measures for Identifying and Predicting the Spread of Complex Contagions

Douglas Guilbeault, Damon Centola
Correspondence to: damon.centola@asc.upenn.edu

This PDF file includes:

Supplementary Methods

Supplementary Notes

Supplementary Figures

Supplementary References

Supplementary Methods

Revising prior measures of bridge width to quantify locally sufficient bridges

Earlier work defined the width of a bridge in terms of the number of overlapping ties between two adjacent neighborhoods on a one-dimensional lattice (1). Let $N[i]$ refer to the inclusive (i.e., closed) neighborhood of node i, defined as the induced subgraph of G including all vertices adjacent to i, along with i. A bridge between neighborhoods $N[i]$ and $N[j]$ was defined as the set of ties between the set of common members of neighborhoods $N[i]$ and $N[j]$, referred to as I_{ij}, and the disjoint set containing the members of $N[i]$ that are not in $N[j]$, referred to as D_{ij}. The width of a bridge was defined as the size of the set of ties from I_{ij} to D_{ij}.

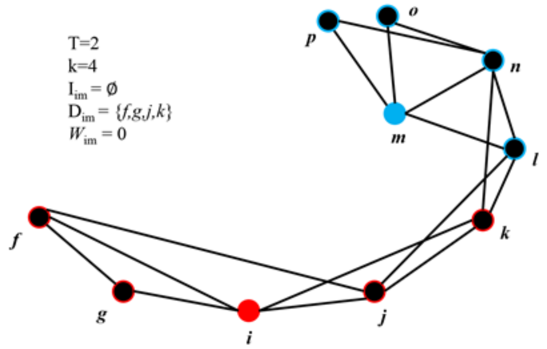

Supplementary Figure 1. Visualizing non-overlapping neighborhoods connected by a sufficiently wide bridge. A schematic display of an edge case for the original bridge width measure based on adjacent neighborhoods. Two connected but non-adjacent neighborhoods are highlighted by different colors: the seed node i is colored red, and members of i's neighborhood are indicated by a red outer ring, while target node m is colored blue, and members of m's neighborhood are indicated by a blue outer ring.

A key limitation of this definition is that it does not generalize beyond adjacent neighborhoods; that is, when $I_{ij} = \emptyset$ (when two neighborhoods have no overlapping members). As a result, this measure has the problem that, even when two neighborhoods are directly connected, it identifies

the size of the bridge between these two neighborhoods as 0, while a complex contagion can spread between them. Supplementary Figure 1 displays one of these edge cases, where two neighborhoods share no overlapping members, even though a complex contagion can spread between them.

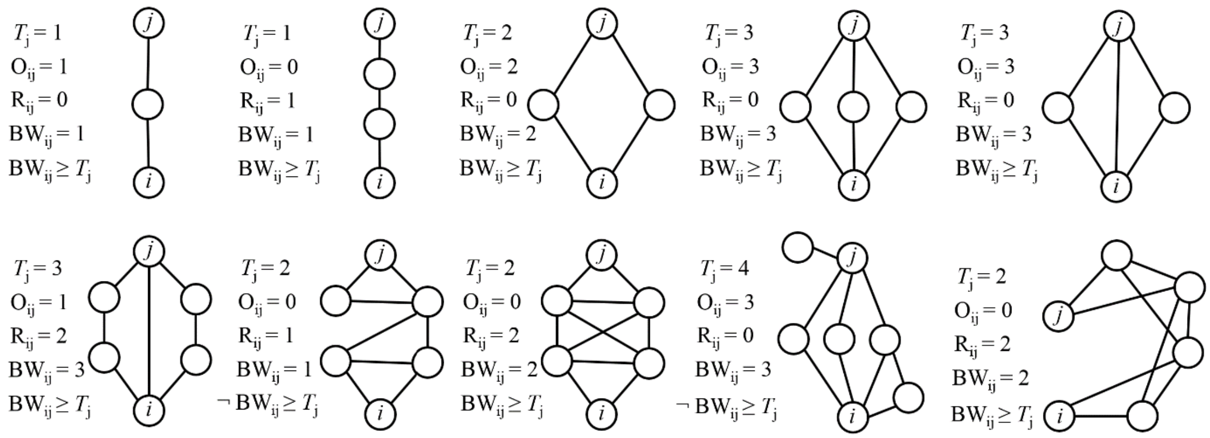

Supplementary Figure 2. Visualizing the measure of bridge width across a variety of neighborhood configurations. These configurations include overlapping and non-overlapping neighborhoods, as well as neighborhoods that are and are not connected by a sufficiently wide bridge. T_j refers to the adoption threshold of target node *j*. O_{ij} refers to the overlap between the inclusive neighborhoods of node *i* and *j*. R_{ij} refers to the size of the reinforcement set connecting the neighborhoods of node *i* and *j*. BW_{ij} refers to the width of the bridge connecting node *i* to node *j*. See the "Methods" section of the main text for the formal definition for each of the terms visualized.

For this reason, we updated the definition of bridge width to account for cases where two neighborhoods are connected, but share no overlapping members, allowing the measure of bridge width to generalize beyond lattices. We develop the following logic for identifying when a sufficient bridge exists that can spread a contagion between two neighborhoods, which share connections but are not necessarily adjacent. Supplementary Figure 2 provides a visual display of how our approach accurately characterizes the width of bridges for both adjacent and non-adjacent but connected neighborhoods. As stated in the main text, for a contagion with an adoption

threshold T_j, a locally sufficient bridge (i.e., a bridge that enables diffusion) exists from node *i*'s neighborhood to node *j*'s neighborhood if (and only if) the following conditions are met.

> Let $N[i]$ refer to the inclusive neighborhood of node *i*, defined as the induced subgraph of *G* including all vertices adjacent to *i*, along with *i*.
>
> Let $E(N[i])$ indicate the edge set of the neighborhood of node *i*, including all ties to *i* within $N[i]$.
>
> Let T_j refer to the adoption threshold of node *j* (i.e., the number or fraction of activated peers that node *j* needs to encounter to adopt).
>
> Let O_{ij} refer to the overlap (intersection) between $N[i]$ and $N[j]$, i.e., $O_{ij} \equiv N[i] \cap N[j]$.
>
> Let D_{ij} refer to the disjoint set of nodes in $N[j]$ that are not in $N[i]$, such that $\forall v(v \in D_{ij} \rightarrow v \in N[j] \wedge v \notin N[i])$
>
> Let R_{ij} refer to the "reinforcement" set of nodes, which consists of the nodes in D_{ij} that are connected to the nodes in $N[i]$. Formally, $\forall v(v \in R_{ij} \rightarrow v \in D_{ij} \wedge |E(N[i]) \cap E(N[v])| \geq 1)$.
>
> Let the bridge between node *i* and *j* be defined as the union of O_{ij} and R_{ij}, i.e. $BW_{ij} \equiv O_{ij} \cup R_{ij}$.
>
> Let the width of the bridge between *i* and *j* be defined as W_{ij}, where $W_{ij} \equiv |BW_{ij}|$ (the cardinality of the bridge between *i* and *j*).
>
> Under the above definitions, the bridge between $N[i]$ and $N[j]$ can support the spread of a contagion – i.e. the bridge is locally sufficient – if W_{ij} is greater than or equal to T_j.
>
> We ascribe every bridge in *G* a binary value indicating whether the bridge is sufficiently wide to enable diffusion. We indicate this binary value in notation by placing sharp brackets around the term for bridge width:

$$[W_{ij}] \equiv \begin{cases} 1 \ if \ W_{ij} \geq T_j \\ 0 \ otherwise \end{cases} \tag{1}$$

The above definition of bridge width can be readily adapted to heterogeneous distributions of thresholds by requiring that R_{ij} contain only of nodes from D_{ij} that can be activated by $N[i]$.

Specifically, this requires that we keep each node x in R_{ij} only if $O_{ix} \geq T_x$ – i.e., if there are enough ties from $N[i]$ to satisfy T_x.

The Add Health Dataset

The Add Health dataset was constructed from an in-school survey, administered to 90,118 students from 84 distinct communities throughout the US in 1994-1995 (2). All network data is publicly available at the following github: https://github.com/drguilbe/complexpaths.[1] The survey was designed to gather data on students' social networks. Each student was given a paper-and-pencil questionnaire and a copy of a roster listing every student in the school and, if the community had two schools, the students were provided with the roster of the "sister" school. Students were asked to "List your closest (male/female) friends. List your best (male/female) friend first, then your next best friend, and so on. (Girls/boys) may include (boys/girls) who are friends and (boy/girl) friends". This dataset was chosen for the purposes of our study because the social networks represent empirically grounded peer networks with significant topological variation.

[1] The data and code for replicating this study can be cited as: Guilbeault, D., & Centola, D. Topological Measures for Identifying and Predicting the Spread of Complex Contagions. drguilbe/complexpaths: First Release (Version v1.0.0). Zenodo. http://doi.org/10.5281/zenodo.5019663 (2021).

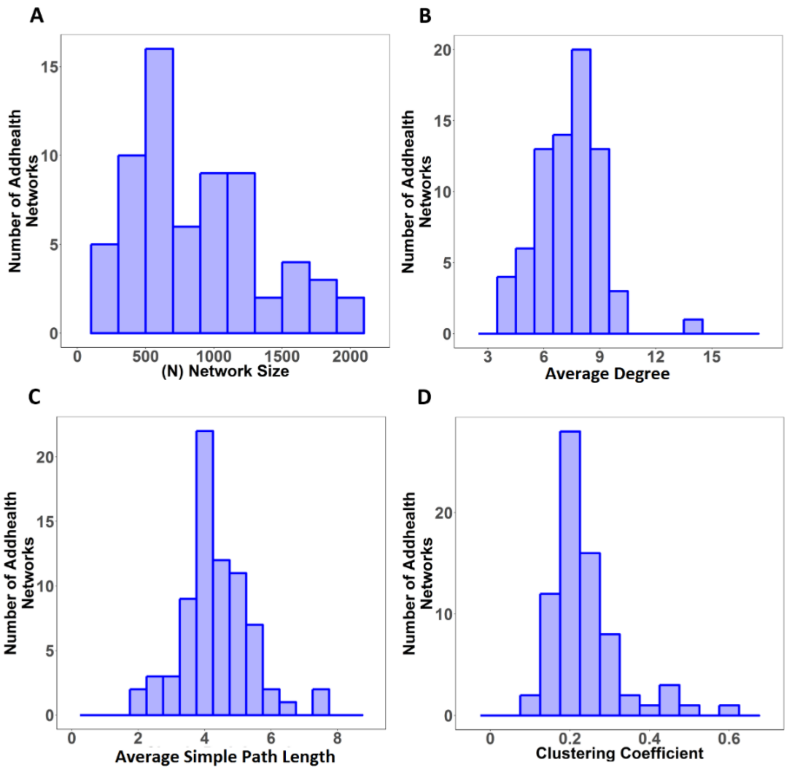

Supplementary Figure 3. Topological properties of the 74 networks used from the Add Health dataset for the empirical simulation analysis. (A) A histogram displaying the distribution of network sizes across all Add Health networks; (B) A histogram displaying the distribution of average degree across all Add Health networks; (C) A histogram displaying the distribution of average simple path length across all Add Health networks; (D) A histogram displaying the distribution of clustering coefficients across all Add Health networks.

For each network in the Add Health dataset, we extracted the largest connected component on which to simulate diffusion and compare different centrality-based seeding strategies. 8 of the 82 networks could not be converted into single connected components, leaving 74 networks available for analysis. The networks varied along several topological dimensions, including size (min. = 25, max. = 2152, $\mu = 814$, $\sigma = 529$), average degree (min. = 3.87, max. = 14, $\mu = 7.32$, $\sigma = 1.63$), average clustering coefficient (min. = .1, max. = .58, $\mu = .27$, $\sigma = .08$), and average simple path length (min. = 1.8, max. = 7.46, $\mu = 4.32$, $\sigma = 1.05$), (Supplementary Figure 3).

Design of the simulated diffusion experiment using the Add Health dataset

For each viable network in the Add Health dataset, we simulated diffusion separately using six seeding strategies: (i) the "complex" seeding strategy, where diffusion was initiated using the seed node identified as having the highest complex centrality; (ii) the "degree" seeding strategy, where diffusion was initiated using the seed node identified as having the highest degree centrality; (iii) the "betweenness" seeding strategy, where diffusion was initiated using the seed node identified as having the highest betweenness centrality; (iv) the "eigenvector" seeding strategy, where diffusion was initiated using the seed node identified as having the highest eigenvector centrality; (v) the "k-core" seeding strategy, where diffusion was initiated using the seed node identified as having the highest coreness; and (vi) the "percolation" seeding strategy, where diffusion was initiated using the seed node identified as having the highest percolation centrality. In all cases, diffusion was initiated with a seeding budget of T_i, where the seed node and $T_i - 1$ of the nodes in its neighborhood were initially activated (if a node's neighborhood contained more than $T_i - 1$, than $T_i - 1$ nodes from this neighborhood were randomly selected as the initial seed set, along with the seed node). In cases where more than one seed node was identified as having the highest centrality according to any of the measures, we randomly selected one of the nodes to be the seed.

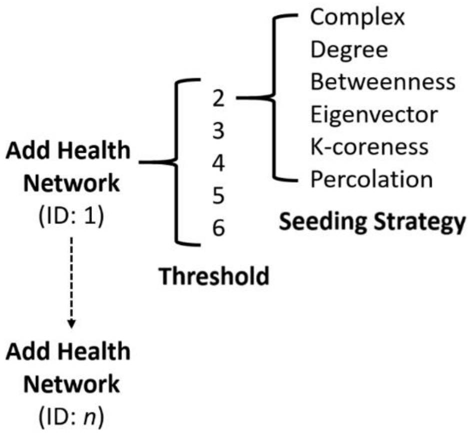

Supplementary Figure 4. A schematic representing the design of the simulated diffusion experiment on the Add Health network dataset. ID., identity of network.

For each network and each seeding strategy, we ran simulations using thresholds ranging from minimal complexity (T_i = 2) to maximal complexity (T_i = 6). (For T_i > 6, we observed minimal to no spreading across all networks.) Specifically, for each seeding strategy, we conducted diffusion trials for each threshold value, $T_i \in \{2,3,4,5,6\}$, on each of the 74 empirical social networks taken from the Add Health dataset (Supplementary Figure 4). For each network and threshold value, we produced six datapoints (representing the number of final adopters) corresponding to the six distinct seeding strategies. Since we tested each simulation strategy on each network for each value of $T_i \in \{2,3,4,5,6\}$, thus producing 30 datapoints for each network, we arrived at 2220 datapoints in total, including all seeding strategies. To average the diffusion outcomes across different homogeneous thresholds on the same network, we first normalize the diffusion outcomes across seeding strategies for each threshold to a 0 to 1 scale using min-max normalization (see "Statistical analysis"). We then average the diffusion outcomes for each seeding strategy across all threshold values for each network, such that each network is

associated with an average normalized number of adopters for each seeding strategy, giving 74 datapoints for each seeding strategy (one for each network), and 444 datapoints in total.

The BSS Microfinance Dataset (Banerjee et al. 2013)

The BSS Microfinance Dataset derives from Banerjee et al. (2013), who collected information about social networks and tracked the adopters of a microfinance program (referred to as the Bharatha Swamukti Samsthe, BSS, program) among all households in 43 distinct villages (3). In each of the villages, the microfinance program was first introduced to the town leaders, who were asked to organize a meeting at which their followers could be informed about the microfinance program and its benefits. Banerjee et al. monitored whether each household in each village adopted the microfinance program overtime, with the ability to link their adoption of the BSS program to each household's position in the village's social network, both with respect to the leaders who seeded the program, and also with respect to the households without leaders that adopted and provided reinforcement for other households to follow suit.

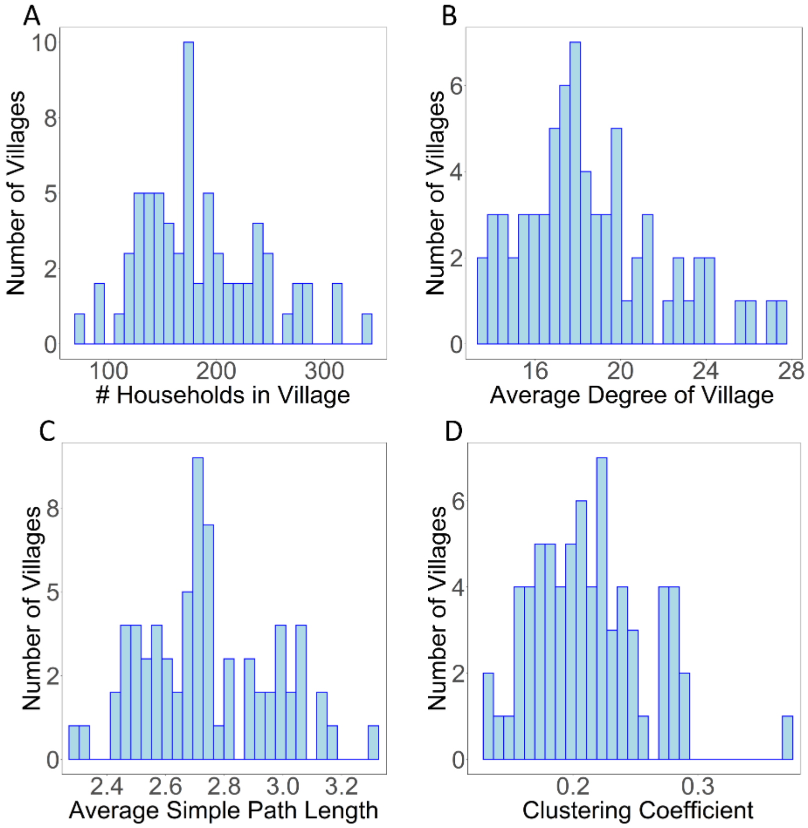

Supplementary Figure 5. Topological properties of the 43 villages used from the BSS dataset on the diffusion of microfinance in rural villages in India collected by Banerjee et al. (2013). (A) A histogram displaying the distribution of village sizes across all villages; (B) A histogram displaying the distribution of average degree across all villages; (C) A histogram displaying the distribution of average simple path length across all villages; (D) A histogram displaying the distribution of clustering coefficients across all villages.

To measure the social network structure of each village, Banerjee et al. administered surveys to each household, which identified social relations across twelve dimensions: those who visit the respondent's home, those whose homes the respondent visits, kin in the village, nonrelatives with whom the respondent socializes, those from whom the respondent receives medical advice, those from whom the respondent would borrow money, those to whom the respondent would lend money, those from whom the respondent would borrow material goods (e.g., kerosene and rice), those to whom the respondent would lend material goods, those from whom the respondent gets

advice, those to whom the respondent gives advice, and those with whom the respondent goes to pray (at a temple, church, or mosque). Banerjee et al. showed how all of these measures can be combined to form a single binary, bidirectional network, where two households are represented as being connected by a single tie if they are connected through at least one of the twelve social dimensions above. A unique strength of this dataset is that Banerjee et al.'s survey also associated each household with a range of demographic and socioeconomic variables – such as the number of beds in the household and whether it has electricity – which can be used as statistical controls when estimating the effect of node centrality on the ability for households to trigger adoption of the BSS program among their network peers.

For each village in the BSS dataset, we extracted the largest connected component on which to simulate diffusion and then use each centrality measure to predict empirical signatures of diffusion as recorded by Banerjee et al. (2013). This preprocessing stage left 43 villages remaining on which we implemented our tests. Supplementary Figure 5 demonstrates that the villages in Banerjee et al.'s study exhibited notable topological variation in terms of size (min. = 75, max. = 341, μ = 186, σ = 56), average degree (min. = 13, max. = 28, μ = 19, σ = 3.31), average simple path length (min. = 2.29, max. = 3.31, μ = 2.74, σ = 0.22), and clustering coefficient (min. = 0.12, max. = 0.36, μ = 0.21, σ = .05). We are grateful to Youm et al. (2021) for making an organized version of the Banerjee et al. (2013) dataset readily available and structured for analysis (4).

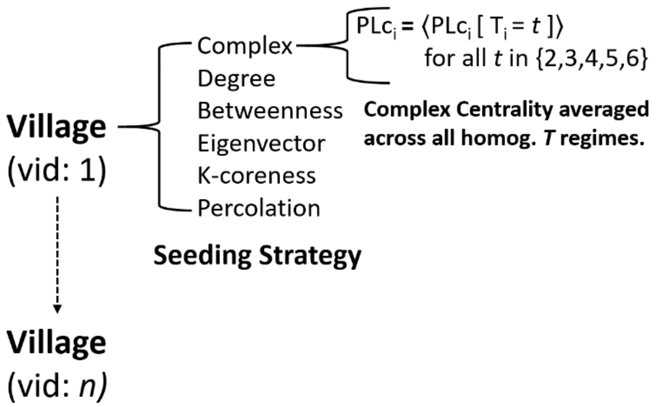

Supplementary Figure 6. A schematic representing the method of analysis for calculating node centrality in each rural village from the Banerjee et al. (2013) dataset. We calculate the average complex centrality of each node across a range of *T* regimes, i.e., where all nodes in the population are ascribed the same T_i from the range, T=2, T=3, T=4, T=5, T=6. All other centrality metrics are independent of T_i and are calculated once on the adjacency matrix of each village. vid, Village identity; homog., homogeneous.

Since it is not possible to directly determine the empirical adoption thresholds that characterized each household's willingness to adopt, we calculate a household's expected complex centrality as its average centrality across a range of adoption thresholds. This methodology is displayed in Supplementary Figure 6. We first simulate diffusion from each household while holding the thresholds of all households constant across a range of absolute adoption thresholds, from $T_i = 2$ to $T_i = 6$. For example, we set the adoption threshold of each household to $T_i = 2$ and then we simulate diffusion when seeding from each possible household. Similar to our Add Health simulation, we adopt a clustered seeding approach. We take the same approach for each T_i from $T_i = 2$ to $T_i = 6$. In each case, when activating a given household as the seed, we set the number of nodes to activate from the seed's neighborhood to $T_i - 1$, identical to our simulated experiments on the Add Health dataset. We then take the average of each household's complex centrality under each value of T_i. As the final step, for each village, we identify the household with the highest

centrality according to each extant centrality measure – degree, eigenvector, betweenness, k-core, and percolation – in addition to identifying the node with the highest average complex centrality. To evaluate our predictions, we compare the ability for each centrality measure to identify influential households, where an empirical measure of household influence is determined by measuring the fraction of a household's neighbors who adopted after the seed household adopted (see Supplementary Table 1 and Supplementary Table 2 for full details on our statistical approach).

Statistical analysis

To average and compare the results of seeding strategies in the case of homogeneous thresholds (where diffusion outcomes vary significantly by T_i for all seeding strategies), we first normalize the diffusion outcomes across all possible nodes for each network and each value of T_i. We use min-max normalizations to standardize diffusion outcomes to a scale from 0 to 1, thus enabling us to average diffusion outcomes across networks with different homogeneous T_i distributions, while preserving the capacity to clearly identify which seeding strategy performed the best. Min-max normalization in implemented using the following formula:

$$z_i = \frac{x_i - \min(x)}{\max(x) - \min(x)}$$

We then average the normalized diffusion outcomes across networks based on seeding strategies to produce an aggregate representation of which seeding strategy performed best across a full range of threshold conditions. Network conditions that involved heterogeneously distributed thresholds did not require normalization.

For the purposes of visualization in Fig. 3, we use min-max normalization to standardize the number of adopters generated across all seeding strategies, holding the network and threshold

constant. This allows us to rank seeding strategies for each network-threshold configuration in terms of their diffusion success, where the seeding strategy with the maximum number of adopters from the set of simulation results across seeding strategies is normalized to 1. Similar to the definition of betweenness centrality (5), we also display the values of complex centrality (on the x-axis) using min-max normalization. We then average the rankings for each seeding strategy across all threshold values for each network, such that each network is associated with an average normalized number of adopters for each seeding strategy, giving 74 datapoints for each seeding strategy (one for each network), and 444 datapoints in total.

To compare the diffusion outcomes of different seeding strategies in our analysis of the Add Health data, we use the nonparametric Wilcoxon Signed Rank Test, which is a paired test that compares the ranks of each seeding strategy, paired at the level of each trial in our simulation experiment (where each trial refers to simulated diffusion with a specific fixed threshold applied to a specific network). That is, when comparing two seeding strategies, this test first determines whether a given seeding strategy gave rise to more adopters than another seeding strategy for each network, under each value of T_i (which is held constant for all nodes in each graph). Across networks, this measure reveals the number of times that one seeding strategy gave rise to more adopters than another strategy (i.e., was ranked higher) across all networks. This test then determines whether the network-level rankings between two conditions are equivalent, or whether they significantly differ. A significant p-value in this case indicates that one seeding strategy gave rise to significantly more adopters on average than another seeding strategy. We use the two-tailed version of the Wilcoxon Signed Rank Test.

In all cases where betweenness centrality (5,6) is calculated in this study, it is calculated based on the standard equation, where the betweenness centrality of a node *v* is given by the expression:

$$g(v) = \sum_{s \neq v \neq t} \frac{\sigma_{st}(v)}{\sigma_{st}}$$

where σ is the total number of shortest paths from node *s* to node *t* and $\sigma_{st}(v)$ is the number of those paths that pass through *v*. As is standard, we normalize betweenness centrality such that $g \in [0,1]$ using min-max normalization.

In all cases where eigenvector centrality (6) is calculated in this study, it is calculated based on the standard equation. For a given graph $G := (V, E)$ with |V| vertices, let $A = (a_{v,t})$ be the adjacency matrix, such that $a_{v,t} = 1$ if vertex *v* is linked to vertex *t*, and $a_{v,t} = 0$ otherwise. Under eigenvector centrality, the relative centrality, *x*, score of vertex *v* can is defined as:

$$x_v = \frac{1}{\lambda} \sum_{t \in M(v)} x_t = \frac{1}{\lambda} \sum_{t \in G} a_{v,t} x_t$$

where *M*(*v*) is the set of neighbors of *v* and λ is a constant. Rearranged, this can be expressed in vector notation as the eigenvector equation: $Ax = \lambda x$.

In all cases where degree centrality is calculated in this study, it is calculated as per standard methodology, simply as the absolute number of connections held by a particular node (6); or, more formally, for a given node *i*, degree centrality is calculated as the number of cells in

the adjacency matrix *A* such that $a_{i,j} = 1$, divided by 2 in the context of unweighted symmetrical ties, to adjust for symmetries in the adjacency matrix.

The measure of clustering coefficient in this work refers to the standard global clustering coefficient (6) based on triplets of nodes. An open triplet refers to three nodes where only two are connected, leaving one pair of nodes in the triplet unconnected (hence open). A closed triplet consists of three nodes that are all connected to each other by undirected ties. The global clustering coefficient uses the proportion of closed to open triplets in a graph as a measure for the average amount of peer reinforcement possible over a whole graph. Formally, it is defined as:

$$C = \frac{\text{number of closed triplets}}{\text{number of all triplets (open and closed)}}$$

As a robustness test, we compare complex centrality seeding to less popular seeding methods that are nevertheless still based on simple path length – i.e., *closeness centrality* and *reach centrality*. Closeness centrality is defined as the reciprocal sum of the length of the shortest (simple) paths between a given node and all other nodes in a graph. Thus, the more central a node, the closer it is to all other nodes (according to the metric of distance supplied by simple path length). Formally, closeness is defined as (7):

$$C(x) = \frac{1}{\sum_y d(y, x)}$$

where $d(y, x)$ is the distance between vertices x and y. When speaking of closeness centrality, it is often represented in its normalized form which represents the average length of the shortest paths

instead of their sum, as given by the previous formula multiplied by $N - 1$, where N is the number of nodes in the graph.

$$C(x) = \frac{N}{\sum_y d(y,x)}$$

This normalization adjustment allows comparisons between nodes of graphs of different sizes.

Reach centrality is a recent measure of centrality that seeks to capture the proportion of other nodes on a graph that are 'reachable' in a diffusion process from a given node (8). The measure, as such, is defined assuming a simple model of contagion, where target nodes only need exposure to one adopter peer in order to adopt themselves. The authors of this measure offer both a local and global measure of reach. Here we focus on comparisons to the local measure of reach, which is defined at the node-level (the level most relevant for the comparison of seeds). The local reaching centrality, $C_{R(i)}$, of node i is the proportion of all nodes in the graph that can be reached from node i via outgoing edges. That is, $C_{R(i)}$ is the number of nodes with a finite positive directed distance from node i divided by $N - 1$ (the maximum possible number of nodes reachable from a given node).

Optimal percolation centrality identifies which nodes are most likely to collapse the largest connected component of a graph – defined in terms of simple paths – when these nodes are removed (9). In practice, percolation centrality amounts to the product of the reduced degree of a node ($k - 1$) and the total reduced degree of all nodes at the optimal distance d. Optimal results are frequently reached when d is either 3 or 4. Optimal percolation centrality was calculated in this paper using the collective influence (CI) algorithm defined by Morone &

Makse (2015; ref. 9). The specifically implementation of the CI algorithm used in this paper is the implementation built into the *influential* package for the statistical programming language R.[2]

The coreness of an algorithm is calculated using the k-shell decomposition algorithm (10). A k-core of a graph G is a maximal connected subgraph of G in which all vertices have degree at least k. Equivalently, it is one of the connected components of the subgraph of G formed by repeatedly deleting all vertices of degree less than k. A vertex has *coreness* c if it belongs to a c-core but not to any $(c+1)$-core.

Supplementary Notes

1. Robustness of the measure of locally sufficient bridges

For the sake of analytic clarity, our main text presents our measure of locally sufficient bridges on graphs subjected to simplifying assumptions along three key dimensions: (*i*) degree uniformity, where every node was given the same number of contacts in their neighborhood, and (*ii*) threshold type, where every node was assigned a fixed absolute adoption threshold referring to the number of adopters to which one needs to be exposed to adopt. Here we relax these assumptions and show that this measure still provides a highly robust predictor of global cascades.

[2] https://cran.r-project.org/web/packages/influential/vignettes/Vignettes.html

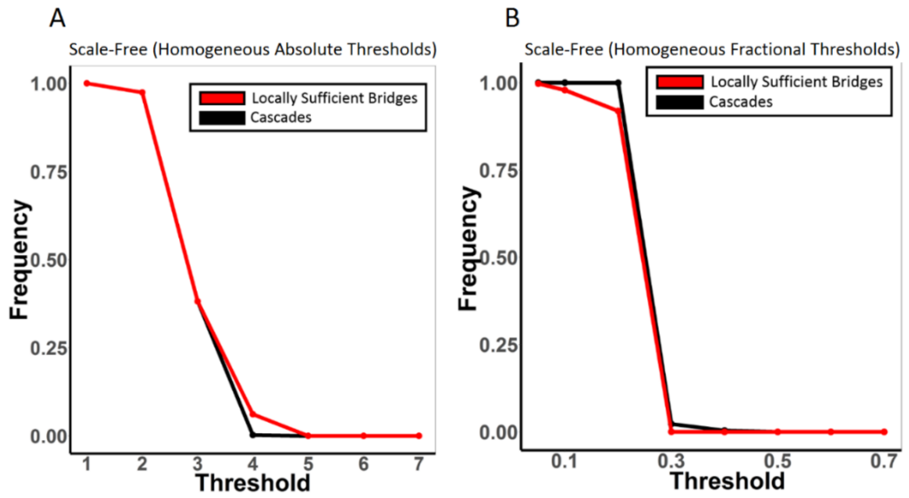

Supplementary Figure 7. Using bridge width to estimate the frequency of global cascades in scale-free networks. Displayed is the relationship between the adoption threshold (T_i) for a contagion (x-axis) and the frequency of seeding events that led to a global cascade, in scale-free networks ($\gamma = 3$, $m = 4$; $p = .5$; $\langle k \rangle = 8$; $N = 1000$), where diffusion is driven by (A) homogeneous absolute thresholds and (B) homogeneous fractional thresholds. The data are averaged across 50 independent simulations for each threshold value.

2. Robustness of the measure of locally sufficient bridges to scale-free networks with homogeneous absolute and fractional thresholds

Here we validate our measure of locally sufficient bridges on scale-free networks to illustrate robustness to degree heterogeneity. We simulated outcomes on 50 randomly generated scale-free networks, produced using Holme and Kim's (12) method of tunable clustering ($\gamma = 3$, $m = 4$; $p = .5$; $N = 1000$). Supplementary Figure 7 illustrates that, even in scale-free networks, our measure of locally sufficient bridges provides a strong fit for the frequency of cascades that arise from simulating diffusion by activating every seed neighborhood in a network – both for homogeneous distributions of absolute thresholds (Supplementary Figure 7*A*) and homogeneous distributions of fractional thresholds.

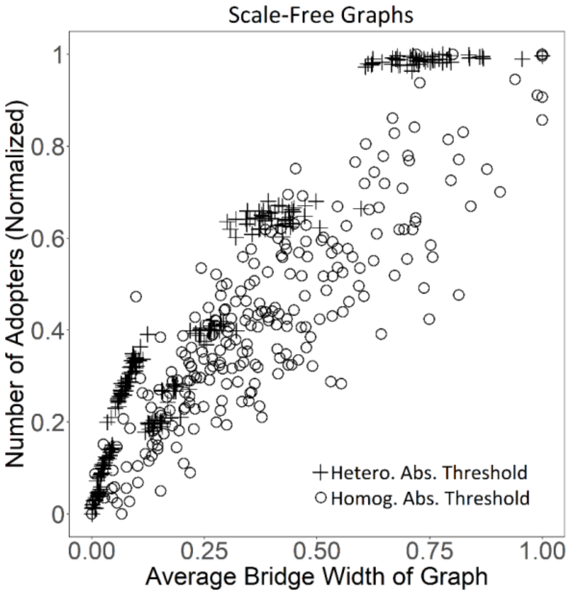

Supplementary Figure 8. The fit between the average bridge width of a graph and the frequency of global cascades in scale-free graphs. The circles indicate this relationship in scale-free networks with homogeneous absolute thresholds (N = 1000; $\gamma = 3$; m = 4; p = .5; T_i = 2, T_i = 3, T_i = 4, T_i = 5, T_i = 6), and the crosses indicate this relationship in scale-free networks with heterogeneous absolute thresholds (N = 1000; $\gamma = 3$; m = 4; p = .5; T_i = [2, 6]). Data are averaged over 50 realizations and replicated over all possible seed neighborhoods. The final number of adopters for each network was standardized using min-max normalization for each threshold condition prior to averaging to facilitate comparisons between threshold regimes and different values of p. Hetero., heterogeneous; Homog., homogeneous; Abs., absolute.

Consistent with this finding, Supplementary Figure 8 shows that the average bridge width of a graph correlates strongly with the average proportion of adopters across all possible seed neighborhoods, both in the case of (i) heterogeneously and (ii) homogeneously distributed absolute thresholds. The average bridge width of a graph is highly effective at predicting diffusion outcomes for scale-free graphs with (i) homogenously distributed absolute thresholds (T_i = 2, T_i = 3, T_i = 4, T_i = 5, T_s = 6; p < .001, r_s = .85), and (ii) heterogeneously distributed absolute thresholds (T_i = [2, 6]; p < .001, r_s = .96).

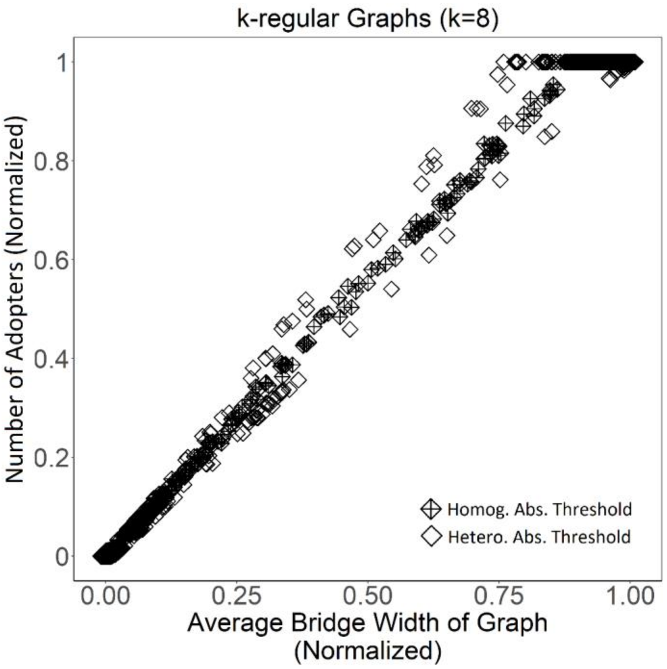

Supplementary Figure 9. The fit between the average bridge width of a graph and the frequency of global cascades in k-regular graphs. The diamonds indicate this relationship in k-regular graphs with homogeneous fractional thresholds (N = 1000; p = [0,1]; ⟨k⟩ = 8; T_i = .1, T_i = .2, T_i = .3, T_i = .4, T_i = .5), and the crossed diamonds indicate this relationship in k-regular graphs with heterogeneous fractional thresholds (N = 1000; p = [0, 1]; ⟨k⟩ = 8; T_i = [.1, .5]). Data are averaged over 50 realizations for each value of p across a range of values ($p = 0$, $p = 2^{-10}$, $p = 2^{-9}$, $p = 2^{-8}$, $p = 2^{-7}$, $p = 2^{-6}$, $p = 2^{-5}$, $p = 2^{-4}$, $p = 2^{-3}$, $p = 2^{-2}$, $p = 2^{-1}$, $p = 2^{0}$), where p indicates the probability of each tie in the network being randomly rewired. The final number of adopters for each network was standardized using min-max normalization for each threshold condition prior to averaging to facilitate comparisons between threshold regimes and different values of p. Slight horizontal jittering is used to reveal overlapping points ($\delta = 0.01$). The data display the results of seeding with all possible seed nodes within each simulated graph. Hetero., heterogeneous; Homog., homogeneous; Abs., absolute.

3. Robustness of correlation for k-regular graphs between average bridge width size and average proportion of adopters

Supplementary Figure 9 shows that the correlation between the average bridge width of a graph and the average proportion of adopters across all possible seeds holds strongly in k-regular graphs (⟨k⟩ = 8), both with (i) homogeneously (T_i = .1, T_i = .2, T_i = .3, T_i = .4, T_i =

.5; $P < .001$, $r_s = .89$) and (ii) heterogeneously distributed thresholds (T_i = [.1, .5]; $P < .001$, $r_s = .98$).

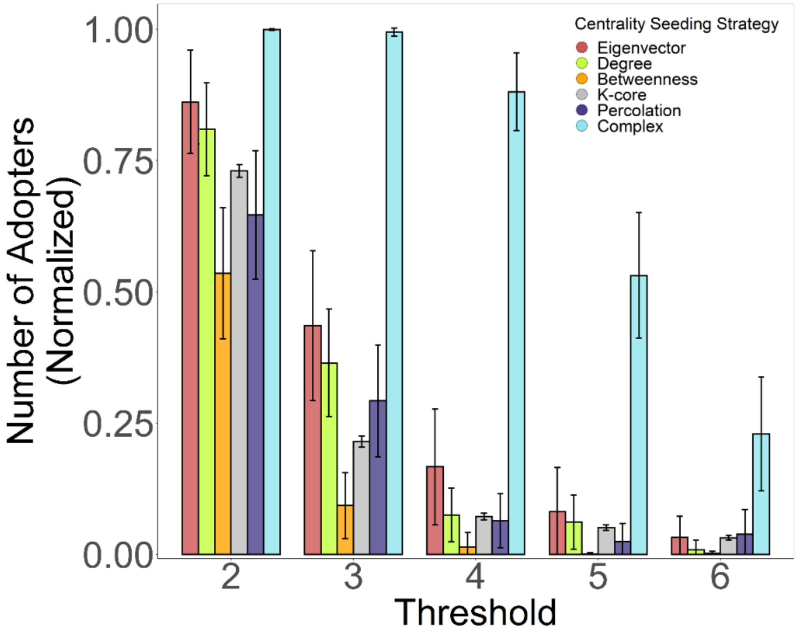

Supplementary Figure 10. Diffusion results for seeding strategies based on node centrality for 74 Add Health networks, differentiated across a range of homogeneously distributed absolute thresholds. The examined range of homogeneously distributed absolute thresholds is displayed along the horizontal axis ($T_i = 2$, $T_i = 3$, $T_i = 4$, $T_i = 5$, and $T_i = 6$). To average the diffusion outcomes on the same graph across different homogeneous threshold conditions, the final number of adopters for each network was standardized using min-max normalization for each threshold condition prior to averaging. This normalization strategy displays the average ranking of each seeding strategy on each network, averaged within each threshold regime. Error bars display 95% confidence intervals.

4. Robustness of Add Health Analysis to Disaggregation by Threshold Regime

Figure 3 in the main text compares centrality-based seeding strategies while associating each seeding strategy with its average diffusion outcome across a range of homogeneous threshold distributions ($T_i = 2$, $T_i = 3$, $T_i = 4$, $T_i = 5$, and $T_i = 6$). Here, we confirm that our results are

robust to comparing seeding strategies in a disaggregated fashion – i.e., by comparing seeding strategies within each homogeneous threshold regime separately. Supplementary Figure 10 shows that seeding with complex centrality produces the highest expected proportion of adopters compared to all other centrality measures, under each homogeneous threshold regime examined.

	Proportion.Adopters		
Predictors	*Estimates*	*CI*	*p*
(Intercept)	0.90	0.84 – 0.95	**<0.001**
Threshold	-0.16	-0.17 – -0.15	**<0.001**
Seeding.Strategy [Degree]	-0.04	-0.08 – 0.01	0.135
Seeding.Strategy [Betweenness]	-0.12	-0.17 – -0.07	**<0.001**
Seeding.Strategy [K-core]	-0.05	-0.10 – -0.01	**0.025**
Seeding.Strategy [Percolation]	-0.08	-0.13 – -0.04	**0.001**
Seeding.Strategy [Complex]	0.10	0.05 – 0.15	**<0.001**
+ Clustered SEs by Network			
Observations	1845		
R^2 / R^2 adjusted	0.419 / 0.417		

Supplementary Table 1. OLS model predicting the proportion of adopters when simulating diffusion using the Add Health data set, while controlling for homogeneous adoption threshold regime and centrality-based seeding strategy, and while clustering standard errors at the network level. Eigenvector centrality is the reference factor in this table for the variable corresponding to seeding strategy. Note: the outcome variable here represents the un-normalized proportion of adopters.

Note, these results are highly robust to comparing seeding strategies using non-normalized diffusion outcomes. Supplementary Table 1 presents the results of an OLS model that predicts the raw proportion of adopters (un-normalized) as a function of seeding strategy, while controlling for adoption threshold and clustering standard errors at the network level. Supplementary Table 1

confirms that seeding with complex centrality is associated with a significant increase in the expected un-normalized proportion of adopters as compared to all other centrality-based measures examined, while holding adoption threshold constant, and while clustering standard errors at the network level (p<0.01, $\beta_{complex}$ = 0.10¸ CI = [0.05,0.15]). For an indication of effect sizes, Supplementary Table 1 shows that – holding threshold constant – seeding with complex centrality is expected to increase the proportion of adopters by 14 percentage points as compared to degree centrality (p<0.01), by 22 percentage points as compared to betweenness centrality (p<0.01), by 15 percentage points as compared to k-core centrality (p<0.01), by 18 percentage points compared to percolation centrality (p<0.01), and by 10 percentage points compared to eigenvector centrality (p<0.01). Furthermore, replicating the same model presented in Supplementary Table 1 while including an additional predictor variable, $\beta_{complex.cent}$, corresponding to the complex centrality associated with each focal seed node identified by each seeding strategy, finds that the complex centrality of a focal seed node is strongly and positively correlated with inducing a higher proportion of adopters, while controlling for seeding strategy and adoption threshold, and while clustering standard errors at the network level (p<0.01, $\beta_{complex.cent.}$ = 0.50¸ CI = [0.47,0.53]).

5. Robustness of complex centrality seeding to network composition and influence model.

Here, we demonstrate the ability for complex centrality to outperform extant measures of centrality in identifying influential nodes across a range of popular influence models. In addition to the complex contagion model, we compare extant seeding strategies in the Independent Cascade (IC) model and the Linear Threshold (LT) model (11). The complex contagion model assumes that all agents require some degree of reinforcement from multiple peers. The IC and LT models, by contrast, provide environments where simple and complex contagion dynamics

can coexist: depending on the model's parameters, some agents may require reinforcement from multiple peers to adopt, whereas other agents in the same population may be able to adopt with exposure to only a single peer, exhibiting the logic of simple contagion. Like the complex contagion model, IC and LT start with an initial set of seeds, and diffusion proceeds in discrete time steps. In IC, when node *i* becomes active in step *t*, it is given only one chance to activate each inactive neighbor *w*, where it succeeds with probability θ. If *i* succeeds, then *w* will become active in $t + 1$. After node *i* attempts to activate *w* at step *t*, node *i* is unable to make any future attempts to activate *w*. In LT, each node *i* is assigned a threshold T_i uniformly at random from the interval [0,1]. Each node in LT is influenced by each neighbor *j* according to a weight b_{ij}, where the sum of weights among *i*'s neighbors is less than or equal to 1. Adoption thresholds in LT thus represent the weighted fraction of *i*'s neighbors that must become active to trigger adoption by *i*. That is, for node *i* at step *t*, node *i* will become active if the summed weight of its active neighbors is greater than or equal to T_i. In all models, diffusion runs until no more activations are possible.

In each influence model, we initiate diffusion from all possible seed nodes, and we use each measure of centrality to identify which of these seed nodes is most successful at triggering diffusion. We compare each centrality across a range of seeding budgets corresponding to the proportion of nodes on a graph that are initially activated as seeds. For each node in a graph, we activate that node and a random subset of its neighbors, where the size of this subset is the size of the seeding budget minus one (for the central node). Given the importance of clustered social influence for complex contagions, we adopt a clustered seeding strategy, such that if the seeding budget exceeds the size of the most central node's neighborhood, we iteratively activate nodes that are directly connected to the neighbors of the most central node until we reach the seeding

budget. Finally, we use each centrality measure to identify which seed node among all possible seed nodes is the most influential. We then compare each centrality measure in terms of its ability to successfully identify influential seed nodes in the spread of complex contagions. We examine the robustness of these results to a suite of both theoretical and empirical topologies.

To further evaluate the efficacy of our centrality measure, we compare our measure against a canonical approach in computer science: a greedy algorithm that simulates diffusion from every possible seed separately and then selects the optimally influential set of nodes with the greatest expected diffusion based on their individual performance (11). We focus our analysis on random scale-free networks with tunable clustering. We begin by comparing different seeding strategies on a large set of simulated scale-free networks, for each influence model: (Model 1) the complex contagion model, using heterogeneous distributions of absolute thresholds, (Model 2) the complex contagion model, using heterogeneous distributions of fractional thresholds, (Model 3) the IC model, and (Model 4) the LT model.

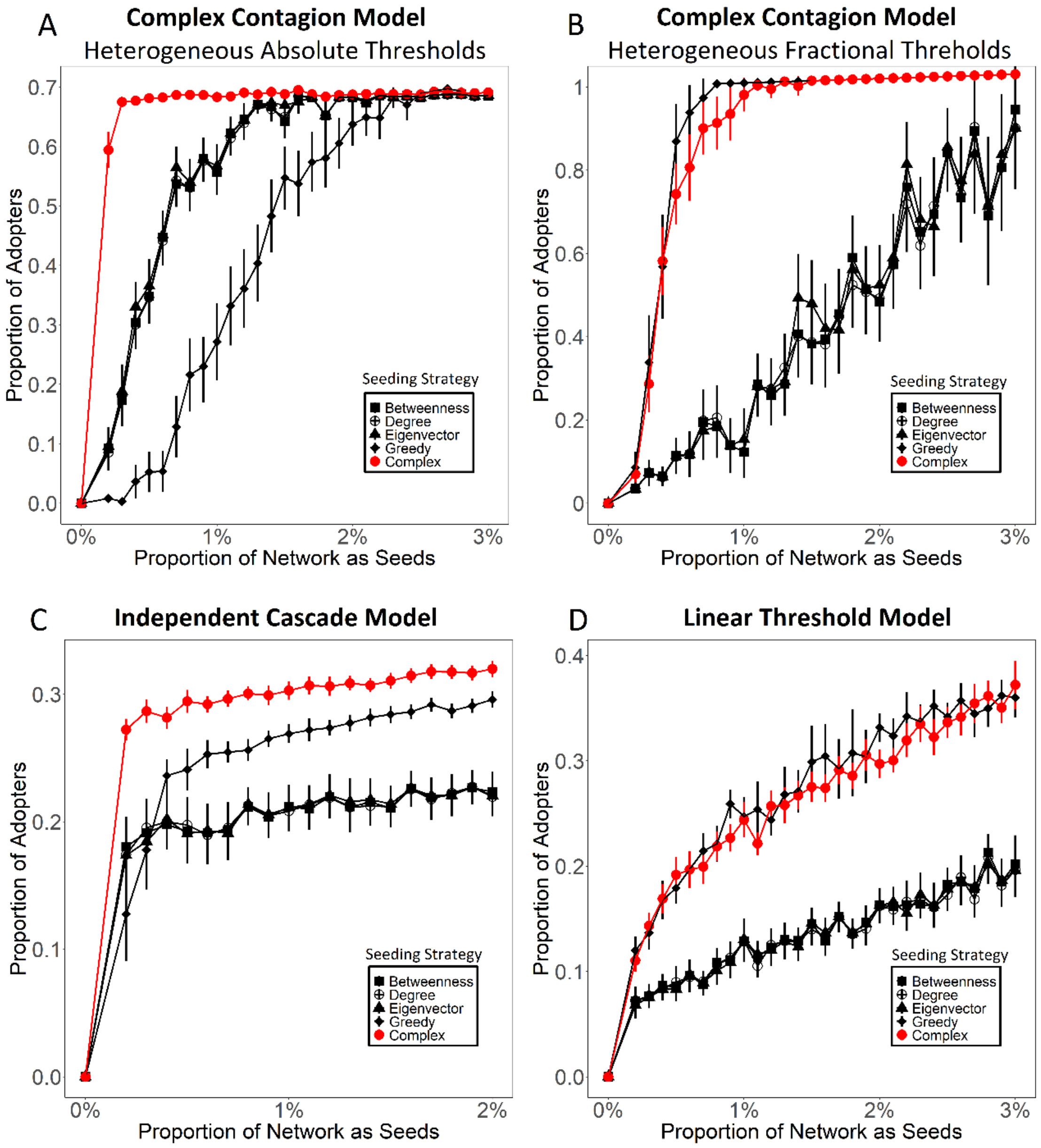

Supplementary Figure 11. Comparing seeding strategies across seeding budgets and influence models. The proportion of adopters averaged over 50 unique scale-free networks (N = 1000; γ = 3; m = 4; p = .5) for seeding strategies based on complex, degree, betweenness, and eigenvector centrality, as well as a greedy sampling algorithm. The first two panels show the success of each seeding strategy for the complex contagion model (A) using heterogeneous absolute thresholds (T_i = [2, 6]) and (B) using heterogeneous fractional thresholds (T_i = [.1,.5]). Second two panels show success of each seeding strategy for (C) the Independent Cascade model (θ = 0.1), and (D) the Linear Threshold model. Error bars display 95% confidence intervals.

Supplementary Figure 11 shows that, in scale-free graphs, selecting seeds with complex centrality leads to strikingly higher levels of diffusion than seeding with standard centrality measures, across a range of threshold conditions, seeding budgets, and influence models. Panel A of Supplementary Figure 11 shows that across seeding budgets from 0.01% to 1%, seeding with complex centrality substantially increases the number of adopters when thresholds are absolute and distributed heterogeneously, as compared to seeding with degree centrality ($n = 60$, $p < .001$, CI=[0.024, 0.184]), betweenness centrality ($n = 60$, $p < .001$, CI=[0.02, 0.18]), and eigenvector centrality ($n = 60$, $p < .001$, CI=[0.017, 0.17]) (Wilcoxon Signed Rank Test, Two-tailed). Further, complex centrality far outperforms the greedy sampling algorithm ($n = 60$, $p < .001$, CI=[0.14, 0.49], Wilcoxon Signed Rank Test, Two-tailed). The greedy sampling algorithm frequently fails to identify seeds that are sufficiently clustered to enable diffusion in the complex contagion model with absolute thresholds.

Panel B of Supplementary Figure 11 shows that these findings replicate in the complex contagion model with heterogeneous fractional thresholds. We find that seeding with complex centrality leads to 100% global adoption with only 1.2% of the network as seeds, whereas the same seeding budget leads to an adoption rate of only 25% on average for degree, betweenness, and eigenvector centrality. It takes over three times as many seeds to generate global adoption using degree ($n = 60$, $p < .001$, CI=[0.40, 0.59]), betweenness ($n = 60$, $p < .001$, CI=[0.39, 0.58]), and eigenvector centrality ($n = 60$, $p < .001$, CI=[0.38, 0.47]), as compared to complex centrality (Wilcoxon Signed Rank Test, Two-tailed). In this model, we find that complex centrality performs as well as the optimal greedy algorithm ($n = 60$, $p = .72$, KS test, Two-tailed).

We find that these results are not limited to the complex contagion model. Complex centrality also outperforms extant seeding strategies in the IC and the LT model. Consistent with

prior work, panel C of Supplementary Figure 11 shows that the greedy algorithm outperforms degree, betweenness, and eigenvector centrality in the IC model (11). However, panel C of Supplementary Figure 11 also shows that in IC, complex centrality outperforms all centrality-based seeding methods and the greedy algorithm. In supplementary analyses, we show that this finding is robust to variation in θ (which specifies the likelihood of successful peer influence in IC). Lastly, panel D of Supplementary Figure 11 shows that in the LT model, complex centrality also significantly outperforms degree, betweenness, and eigenvector centrality seeding methods, equivalent to the optimal greedy algorithm.

6. Robustness of complex centrality seeding to scale-free networks with varying levels of clustering.

To test the robustness of our results to a wide range of scale-free networks that vary in terms of average clustering coefficient, we generated scale-free networks using Holme and Kim's (10) method of tunable clustering. The parameter *p* in this approach determines the probability of adding a triangle after adding a random edge to the network (see "Statistical analysis"). The results of our seeding analysis on scale-free networks in the main text (Fig. 2) used the following parameter settings ($\gamma = 3$, $m = 4$; $p = .5$; $N = 1000$) to produce networks with an average clustering coefficient (C) of .27. Here we demonstrate the robustness of our results to scale-free networks generated with significantly lower and significantly higher levels of clustering.

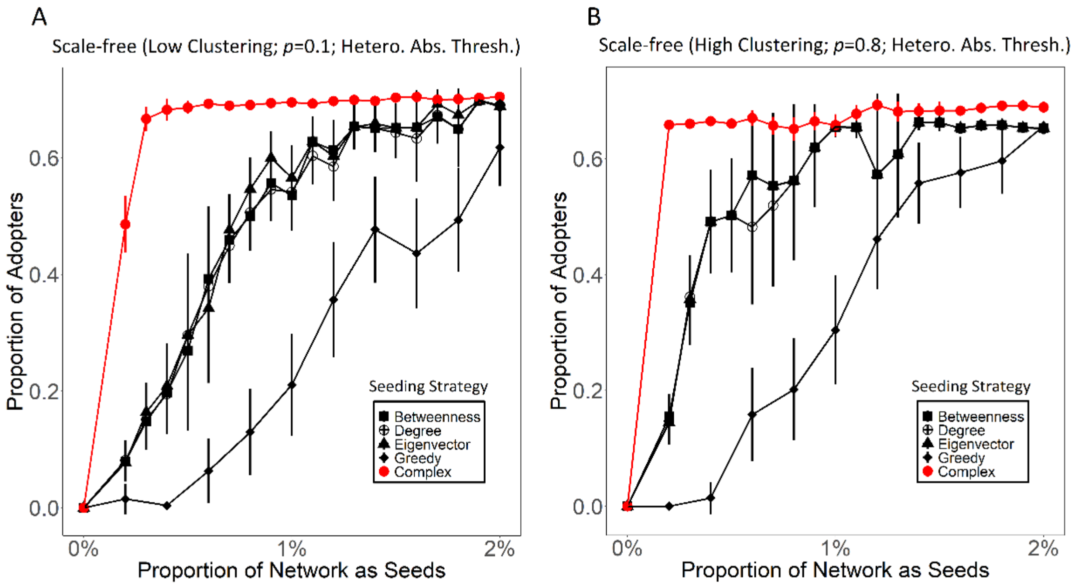

Supplementary Figure 12. Comparing seeding strategies across seeding budgets, while varying the level of clustering in scale-free networks. The proportion of adopters averaged over 100 unique scale-free networks for seeding strategies based on node centrality (complex, degree, betweenness, and eigenvector) and a greedy sampling algorithm, under different levels of clustering and seeding budgets (i.e., the proportion of nodes in the network that are initially activated as seeds). All panels present diffusion results with heterogeneous absolute thresholds (T_i = [2, 6]). (A) Scale-free parameters: $p = .1$, $\gamma = 3$, $m = 4$, $p = .1$, $N = 1000$, clustering coefficient = .07; (B) Scale-free parameters: $p = .8$, $\gamma = 3$, $m = 4$, $N = 1000$, $C = .38$. Error bars display 95% confidence intervals. Hetero., heterogeneously distributed; Hetero. Abs. Thresh., heterogeneous distribution of absolute adoption thresholds.

Supplementary Figure 12 shows that the same results reported for Fig. 2 equally hold for scale-free networks with low and high levels of clustering. Panel A of Supplementary Figure 12 shows that when scale-free networks are generated with low levels of clustering ($p = .1$, $\gamma = 3$, $m = 4$, $p = .1$, $N = 1000$, C = .07), complex centrality produces significantly greater levels of diffusion across a range of seeding budgets, compared to seeding with betweenness centrality ($p < .001$), degree centrality ($p < .001$), eigenvector centrality ($p < .001$), and a greedy sampling algorithm ($p < .001$) (Wilcoxon Signed Rank Test, Two-tailed). Similarly, panel B of Supplementary Figure 12 shows that when scale-free networks are generated with high levels of

clustering (p = .8, γ = 3, m = 4, p = .8, N = 1000, C = .38), complex centrality produces significantly greater levels of diffusion across a range of seeding budgets, compared to seeding with betweenness centrality (p<0.001), degree centrality (p < .001), eigenvector centrality (p < .001), and a greedy sampling algorithm (p < .001) (Wilcoxon Signed Rank Test, Two-tailed).

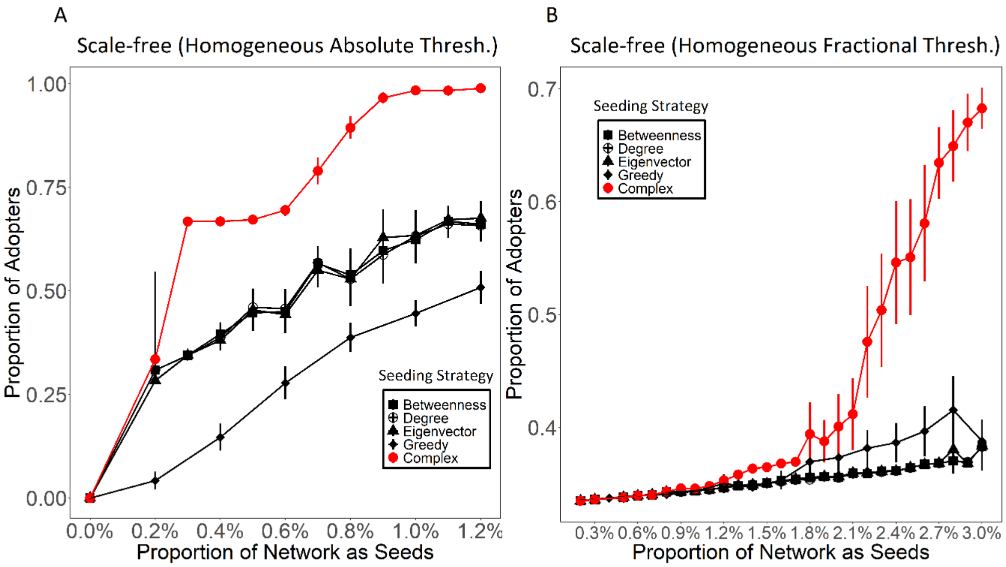

Supplementary Figure 13. Comparing seeding strategies across seeding budgets in scale-free networks with homogeneous thresholds, absolute and fractional. The proportion of adopters averaged over 50 unique scale-free networks (N = 1000; γ = 3; m = 4; p = .5) for seeding strategies based on complex, degree, betweenness, and eigenvector centrality, as well as a greedy sampling strategy. Success of seeding strategies are shown in the complex contagion influence model (A) when using homogeneous absolute thresholds (T_i = 2, T_i = 3, T_i = 4, T_i = 5, T_i = 6) and (B) when using homogeneous fractional thresholds (T_i = .1, T_i = .2, T_i = .3, T_i = .4, T_i = .5). Error bars display 95% confidence intervals. Thresh., adoption thresholds.

7. Robustness to homogeneous absolute and fractional thresholds in the scale-free graphs

In Supplementary Figure 13, we present our results on scale-free graphs with heterogeneous threshold distributions, which we foreground in the main text because they capture expected heterogeneity in a population. Here in Supplementary Figure 13 we show that our seeding results also hold in graphs with homogeneous distributions of absolute and fractional thresholds in

scale-free networks (N = 1000; γ = 3; m = 4; p = .5). Panel A of Supplementary Figure 13 shows that seeding with complex centrality leads to significantly greater diffusion than extant seeding strategies in scale-free graphs with homogeneous absolute thresholds; and panel B of Supplementary Figure 13 shows that seeding with complex centrality leads to significantly greater diffusion than seeding with extant seeding strategies in scale-free graphs with homogeneous fractional thresholds.

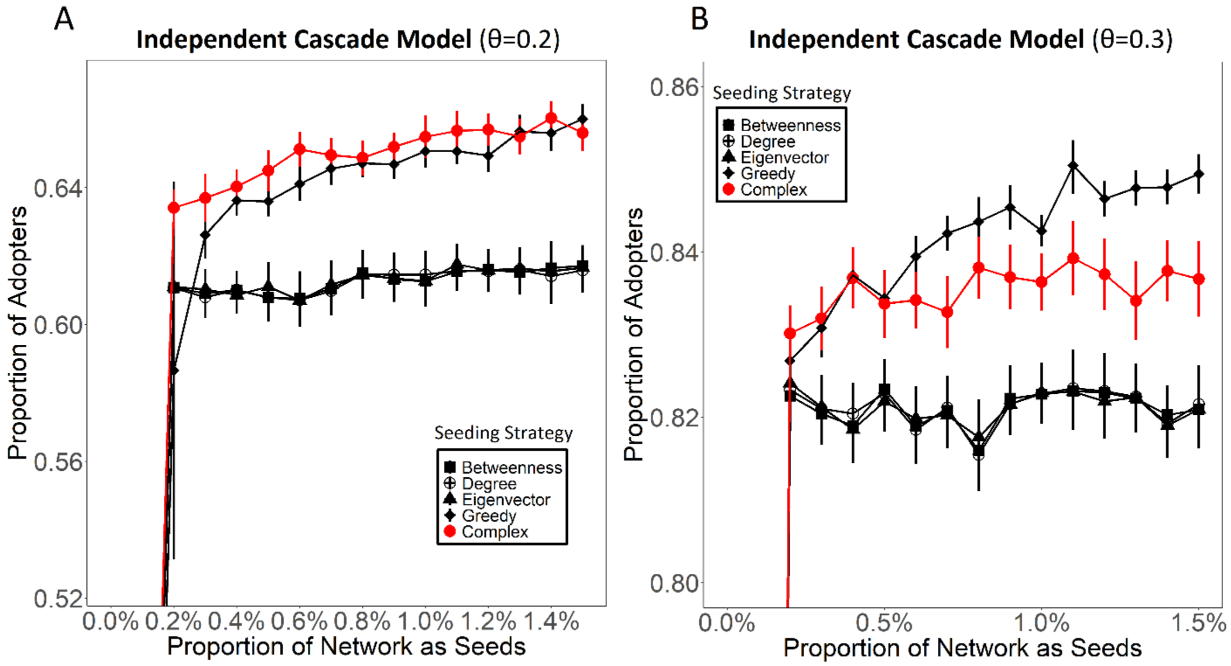

Supplementary Figure 14. Comparing seeding strategies in the Independent Cascade model, while varying θ, the probability that a given peer interaction in the network will enable diffusion. The proportion of adopters in the Independent Cascade model averaged over 30 unique scale-free networks (γ = 3, m = 4, p = .5, N = 1000) for seeding strategies based on node centrality (complex, degree, betweenness, and eigenvector) and the greedy algorithm. (A) θ=0.2; (b) θ=0.3. Error bars display 95% confidence intervals.

8. Robustness to varying the probability of adoption in the independent cascade model

Supplementary Figure 14 shows that complex centrality outperforms all other centrality-based seeding strategies across a range of θ values in the IC model (where θ refers to the probability

that any interaction between an adopter and nonadopter in the network will permit diffusion). In the main results, we reported simulations of IC with θ = 0.1, where complex centrality was shown to outperform all other centrality measures and the greedy algorithm. Complex centrality is the most successful in this environment, because when θ is low, reinforcing ties among peers can be essential for enabling a cascade. As θ increases, contagion dynamics become "simpler" in that single tie encounters become more likely to trigger adoption without peer reinforcement. Panel A of Supplementary Figure 14 shows that when θ = 0.2, complex centrality continues to outperform all standard centrality-based measures, as well as the greedy algorithm. Panel B of Supplementary Figure 14 shows that when θ = 0.3, contagion dynamics become increasingly simple, and we find that complex centrality still outperforms all other standard centrality measures, but the greedy algorithm starts outperforming complex centrality. However, Supplementary Figure 14 also illustrates that when θ increases, all seeding strategies generate high levels of diffusion (reaching between 96 – 100% network saturation) and are thus only minimally distinguished in practice. By contrast, when θ is low, the choice of seeding strategy can result in qualitatively different levels of diffusion. It is in these more sensitive environments that complex centrality is by far the most impactful approach, leading to a 15% (percentage-point) increase in the proportion of adopters compared to the greedy algorithm, and to a 37% (percentage-point) increase in the proportion of adopters compared to standard centrality-measures (Fig. 2).

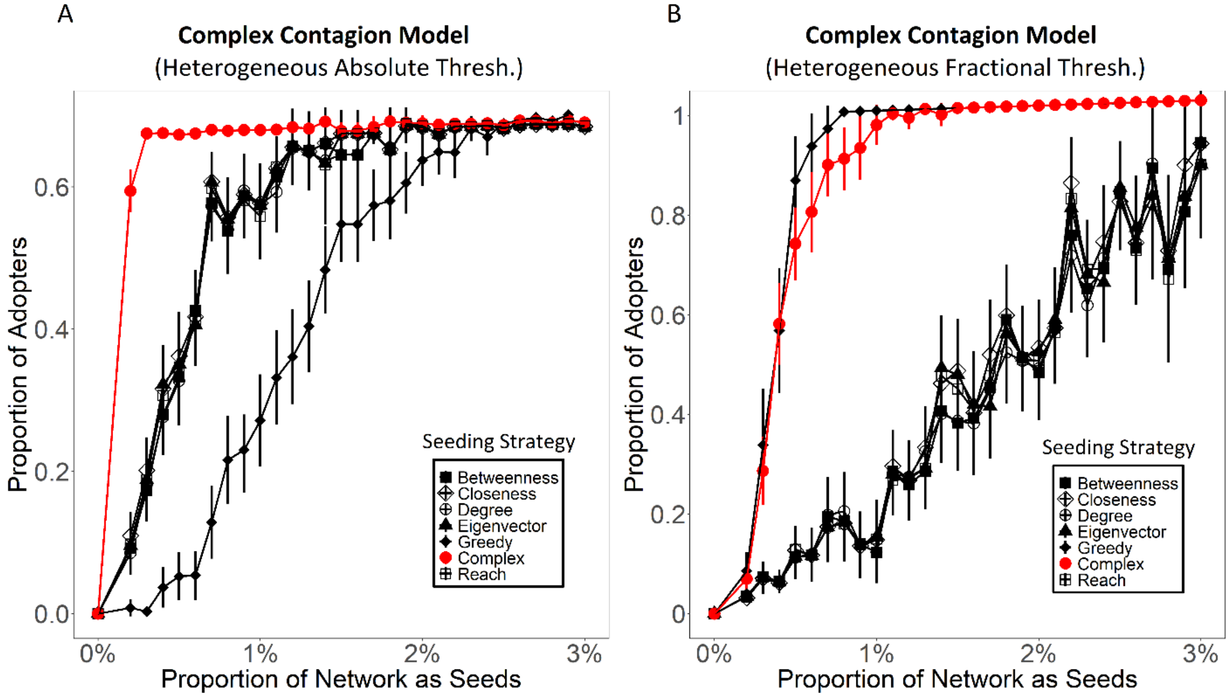

Supplementary Figure 15. Comparing seeding strategies across seeding budgets, including uncommon strategies. The proportion of adopters averaged over 100 unique scale-free networks (N = 1000; $\gamma = 3$; m = 4; p = .5) for seeding strategies based on complex, degree, betweenness, eigenvector, closeness, and reach centrality, as well as a greedy sampling strategy. Success of seeding strategies are shown in the complex contagion influence model (A) when using heterogeneous absolute thresholds (T_i = [2, 6]) and (B) when using heterogeneous fractional thresholds (T_i = [.1,.5]). Error bars display 95% confidence intervals. Thresh., adoption thresholds.

9. Robustness of complex centrality seeding in comparison to closeness and reach centrality

For succinctness in the main text, we report the advantages of seeding with complex centrality in comparison to the most popular centrality-based seeding strategies based on simple path length – i.e., degree, betweenness, and eigenvector centrality. Here we show that complex centrality also substantially outperforms established centrality measures that are less frequently used in seeding. Supplementary Figure 15 shows that, across a range of seeding budgets, complex centrality triggers substantially greater diffusion than closeness centrality (5) and reach centrality (6) in

scale-free networks with heterogeneous absolute thresholds, as well as scale-free networks with heterogeneous fractional thresholds.

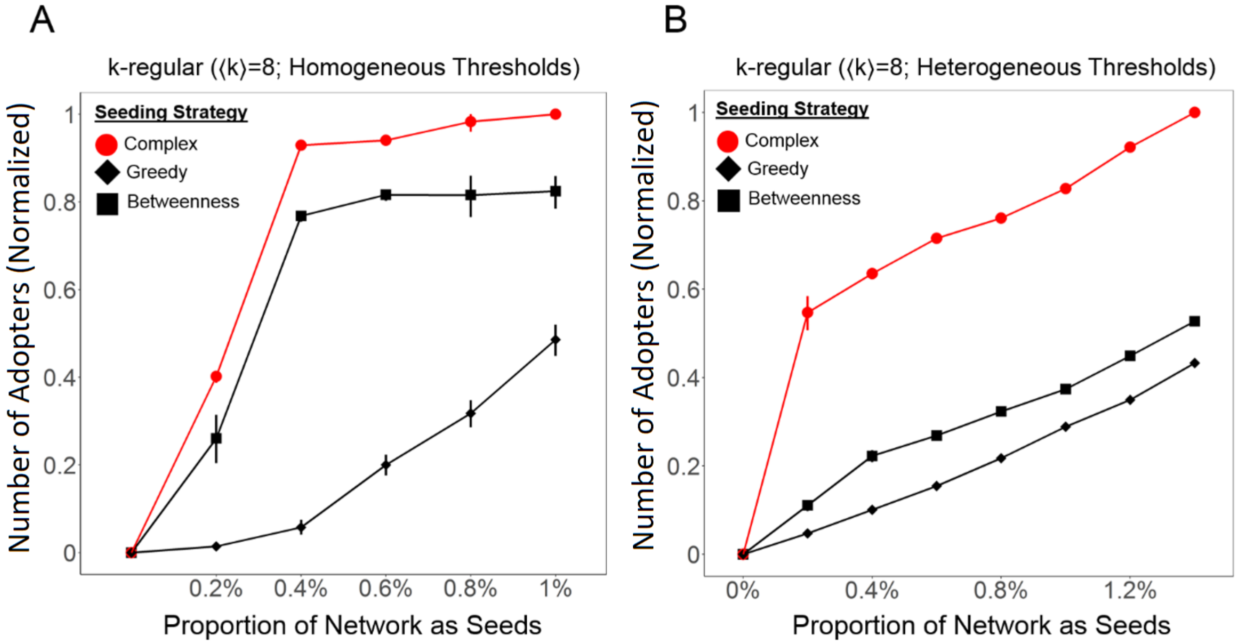

Supplementary Figure 16. Comparing seeding strategies across seeding budgets in k-regular networks with homogeneous and heterogeneous adoption thresholds. The proportion of adopters averaged over a continuum of k-regular networks (N = 1000, ⟨k⟩ = 8), with over 30 unique networks produced at each level of randomization controlled by p (p = 0, $p = 2^{-10}$, $p = 2^{-9}$, $p = 2^{-8}$, $p = 2^{-7}$, $p = 2^{-6}$, $p = 2^{-5}$, $p = 2^{-4}$, $p = 2^{-3}$, $p = 2^{-2}$, $p = 2^{-1}$, $p = 2^{0}$). p indicates the probability of each tie in the network being randomly rewired. Diffusion results are compared for different seeding strategies based on node centrality (i.e. complex and betweenness) and a greedy sampling algorithm, under different threshold conditions and seeding budgets. The final number of adopters for each network was standardized using min-max normalization for each threshold condition prior to averaging to facilitate comparisons between threshold regimes and different values of p. (A) Success of seeding strategies in k-regular graphs with homogenous absolute thresholds (T_i = 2, T_i = 3, T_i = 4, T_i = 5, T_i = 6); (B) using heterogeneous absolute thresholds (T_i = [2, 6]). Error bars display 95% confidence interval.

10. Robustness of complex centrality seeding to k-regular graphs.

In Supplementary Figure 8, we present our results on scale-free graphs with nonuniform degree distributions, which are of relevance to seeding in extant empirical social networks that normally

have nonuniform degree distributions. Here we show that our seeding results also hold in graphs with a uniform degree distribution (i.e., k-regular graphs), which are regularly employed in structured social contexts (14,15). Supplementary Figure 16 shows that seeding with complex centrality leads to significantly greater diffusion than seeding with extant seeding strategies in k-regular graphs of varying levels of randomness in tie distribution. Panel A of Supplementary Figure 16 shows that across a range of seeding budgets, seeding with complex centrality in k-regular graphs with homogeneous absolute thresholds substantially increases the number of adopters, as compared to betweenness centrality ($p < .001$) and the greedy seeding algorithm examined in the main text ($p < .001$) (Wilcoxon Signed Rank Test, Two-tailed) (Note: there is no degree centrality and no meaningful eigenvector centrality in k-regular graphs). The same result for k-regular graphs with heterogeneously distributed thresholds is shown in panel B of Supplementary Figure 16, where seeding with complex centrality triggers much higher levels of adoption than seeding with betweenness ($p < .001$) and the greedy seeding algorithm ($p < .001$) (Wilcoxon Signed Rank Test, Two-tailed).

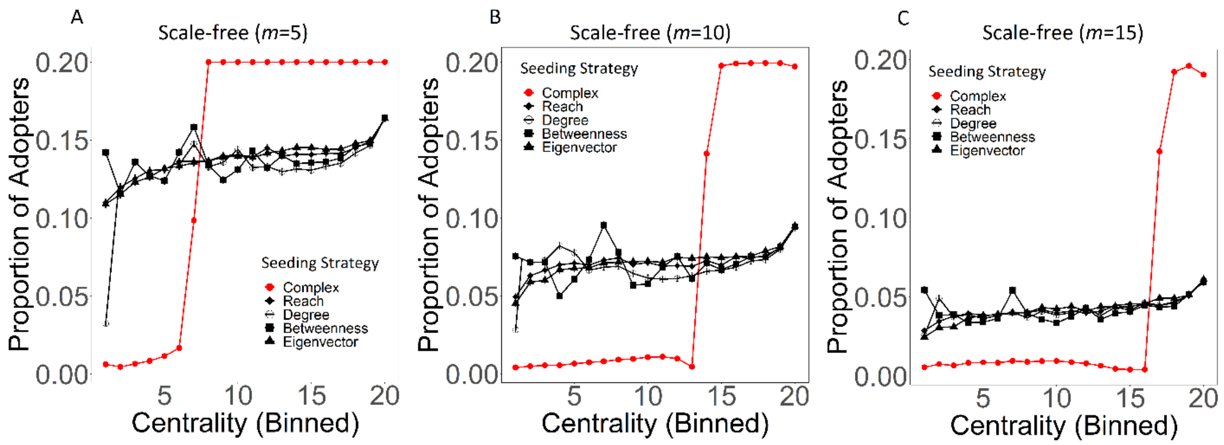

Supplementary Figure 17. Comparing seeding strategies in conventionally generated scale-free networks. The proportion of adopters averaged over 20 unique conventional scale-free networks (N = 1000) across different values of m

(specifying the number of ties to add with each new node in the graph) for seeding strategies based on complex, reach, degree, betweenness, and eigenvector centrality. Success of seeding strategies are shown in the complex contagion influence model using heterogeneous fractional thresholds (T_i = [.1,.5]). Results are aggregated across a range of seeding budgets, from 2 to 8 nodes as initial seeds. (A) with m=5; (B) with m=10; (C) with m=15.

11. Robustness to conventionally-generated scale-free graphs

Here, we confirm that our theory is consistent with scale-free networks generated by the conventional algorithm from the Barabási-Albert model (13). Supplementary Figure 17 illustrates that nodes with the highest complex centrality consistently led to a significantly higher proportion of adopters than extant centrality measures (p<0.001 for each pairwise comparison, Wilcoxon Signed-Rank Test) in conventionally generated scale-free networks, across a range of m values, where m specifies the number of ties to add to the graph when adding a new node in the network generation algorithm. Results are aggregated across a range of seeding budgets, from 2 to 8 nodes as initial seeds.

12. Statistical details on the structural position of nodes with high complex centrality

Figures 3*B-G* in the main text show that seeds with the highest complex centrality have putatively low centrality according to popular measures of node centrality based on simple path length. Here, we explicitly detail the statistical comparisons captured by figures 3*B-G*.

Figure 3*B* shows that seeds identified with the highest complex centrality have significantly lower betweenness centrality ($\langle g(i) \rangle$ = .01) than nodes with the highest degree centrality ($\langle g(i) \rangle$ = .04, n = 148, p < .001, CI=[-0.02,-0.009]), eigenvector centrality ($\langle g(i) \rangle$ = .02, n = 148, p < .001, CI=[-0.02, -0.003]), percolation centrality ($\langle g(i) \rangle$ = .03, n = 148, p < .001,

CI=[-0.02,-0.01]), and betweenness centrality (⟨*g(i)*⟩ = .08, *n* = 148, *p* < .001, CI=[-0.05,-0.03]) (Wilcoxon Signed Rank Test, Two-tailed).

Figure 3*C* shows that seeds with the highest complex centrality have significantly lower degree (⟨k⟩ = 12) than nodes with the highest degree (⟨k⟩ = 24, *n* = 148, *p* < .001, CI=[-13.7,-10.3]), betweenness (⟨k⟩ = 17, *n* = 148, *p* < .001, CI=[-6.4,-2.6]), eigenvector (⟨k⟩ = 23, *n* = 148, *p* = .05, CI=[-12.3,-7.57]), and percolation centrality (⟨k⟩ = 22, *n* = 148, *p* < .001, CI=[-12.3,-8.6]) (Wilcoxon Signed Rank Test, Two-tailed).

Figure 3*D* shows that seeds with the highest complex centrality have significantly lower eigenvector centrality (⟨x_v⟩ = .1) than nodes with the highest degree (⟨x_v⟩ = .2, *n* = 148, *p* < .001, CI=[-0.17,-0.13) and eigenvector centrality (⟨x_v⟩ = .27, *n* = 148, *p* < .001, CI=[-0.22,-0.20]) (Wilcoxon Signed Rank Test, Two-Tailed). Figure 3*E* shows that seeds with the highest complex centrality have significantly lower percolation centrality (⟨*ci*⟩ = .41) than nodes with the highest degree (⟨*ci*⟩ = .86, *n* = 148, *p* < .001, CI=[-0.53,-0.42]), betweenness (⟨*ci*⟩ = .67, *n* = 148, *p* < .001, CI=[-0.37,-0.2]), eigenvector (⟨*ci*⟩ = .77, *n* = 148, *p* < .001, CI=[-0.45,-0.22]), and percolation centrality (⟨*ci*⟩ = 1.0, *n* = 148, *p* < .001, CI=[-0.61,-0.55]) (Wilcoxon Signed Rank Test, Two-Tailed; percolation centrality is normalized using min-max normalization).

Note that nodes with the highest complex centrality do not have higher degree, betweenness, eigenvector, or percolation centrality than nodes with the highest k-coreness. Yet, Figure 3*F* shows that nodes with the highest complex centrality are nevertheless structurally distinct from nodes with the highest k-coreness. Figure 3*F* shows that seeds with the highest complex centrality have the lowest k-core centrality (⟨*c*⟩ = .29) compared to all other measures, including nodes with the highest k-core (⟨*c*⟩ = 1.0, *n* = 148, *p* < .001, CI=[-0.83,-0.61]), degree

(⟨*c*⟩ = .74, *n* = 148, *p* < .001, CI=[-0.58,-0.36]), betweenness (⟨*c*⟩ = .44, *n* = 148, *p* < .05, CI=[-0.19,-0.03]), eigenvector (⟨*c*⟩ = .90, *n* = 148, *p* < .001, CI=[-0.8,-0.51]), and percolation centrality (⟨*c*⟩ = .63, *n* = 148, *p* < .05, CI=[-0.4,-0.18]) (Wilcoxon Signed Rank Test, Two-Tailed).

Lastly, Fig. 3*G* illustrates the nodes with the highest complex centrality have significantly higher complex centrality than the influencers identified by all other measures (*p*<0.001 for all pairwise comparisons, Wilcoxon Signed Rank Test, Two-Tailed).

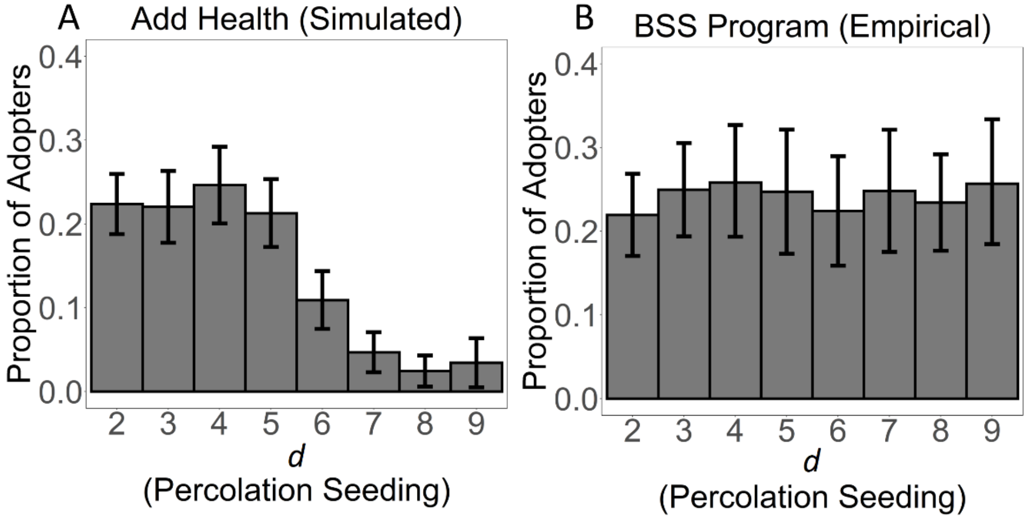

Supplementary Figure 18. Comparing the diffusion outcomes of seeding with optimal percolation centrality, across different values of the hyperparameter *d*. Diffusion outcomes are shown for (A) simulated diffusion outcomes in the Add Health data set (averaged for T_i=2, T_i=3, T_i=4, T_i=5, T_i=6), and for (B) empirical diffusion outcomes of the BSS microfinance program in the Banerjee et al. (2013) dataset (3). Error bars display 95% confidence intervals.

13. Robustness to hyperparameter d in seeding with percolation centrality

The optimal percolation centrality method – also known as the collective influence algorithm (CI) – defined by Morone and Makse (2015) is tuned by a hyperparameter, *d*, which specifies the

distance from the focal node (i.e., the number of steps along simple paths) within which alter nodes will be assessed in terms of their reduced degree. Our main results presented in figure 3 and 4 assign *d* the standard and default value of *3* steps. For thoroughness, we show here that altering *d* does not lead to any significant improvements in the overall proportion of adopters induced by seeds selected by percolation centrality, either in simulated diffusion outcomes on the Add Health dataset (panel A of Supplementary Figure 18) or in the empirical diffusion of the BSS microfinance program as measured by Banerjee et al. (2013; ref. 3) (panel B of Supplementary Figure 18). Diffusion is measured in the BSS program here by examining all potential adopters, and by examining only those outcomes observed when seeding from leader households that agreed to aid in the diffusion of the program (3).

Seeding with Leader Households
(Predicting Adoption by any Network Neighbor)

	Probability.Adoption.All.Neighbors		
Predictors	*Estimates*	*CI*	*p*
(Intercept)	-0.74	-1.03 – -0.45	**<0.001**
Seeding.Strategy [Betweenness]	0.00	-0.04 – 0.04	0.908
Seeding.Strategy [Complex]	0.07	0.03 – 0.11	**0.001**
Seeding.Strategy [K-core]	0.03	-0.01 – 0.07	0.173
Seeding.Strategy [Degree]	0.01	-0.03 – 0.05	0.728
Seeding.Strategy [Eigen]	-0.00	-0.04 – 0.04	0.962
Seeding.Strategy [Percolation]	0.02	-0.02 – 0.06	0.370
nrooms	-0.02	-0.04 – 0.01	0.147
nbeds	0.07	0.03 – 0.10	**<0.001**
electricity	0.08	0.04 – 0.11	**<0.001**
latrine	0.07	0.05 – 0.09	**<0.001**
nroomscapita	0.30	0.19 – 0.41	**<0.001**
nbedscapita	-0.38	-0.56 – -0.20	**<0.001**
trimester	0.07	0.04 – 0.11	**<0.001**
Village Fixed Effects	✓	✓ ✓	✓
Observations	279		
R^2 / R^2 adjusted	0.766 / 0.713		

Supplementary Table 2. OLS model using each centrality measure to predict the fraction of each seed household's network neighborhood that adopted the BSS program (using 'leader' households only), while controlling for all socioeconomic variables included in Banerjee et al.'s (2013) survey, with additional fixed effects at the village-level. The intercept identifies the expectation when randomly selecting leader seed households. The results are robust to varying the seeding strategy used as the referent strategy for the intercept.

14. Robustness of BSS diffusion model to statistical controls

In this final supplementary section, we illustrate that the results presented in figure 4 are robust to a myriad of statistical tests and socioeconomic control variables. Supplementary Table 2

displays the fit of an OLS model that uses each centrality measure to predict the fraction of each seed household's neighborhood that adopted the BSS program (using 'leader' households only), while controlling for all socioeconomic variables included in Banerjee et al.'s (2013) survey, with additional fixed effects at the village level (3). The intercept identifies the expectation when randomly identifying leader households. The results are robust to varying the seeding strategy used as the referent strategy for the intercept. We see that, even when subject to all of the above controls, only seeding with complex centrality is associated with a significant increase in the fraction of adopters relative to randomly selected leader households (p=0.001, $\beta_{complex}$ = 0.07¸CI=[0.03, 0.11]). This effect still holds when clustering standard errors at the village level (p<0.05, $\beta_{complex}$ = 0.07). Overall, the above model accounts for 76% of the variance in the ability for leader households to trigger adoption of the BSS program among their network neighbors.

Seeding with any Household
(Predicting Adoption by any Network Neighbor)

	**Probability.Adoption.All.Neighbors**		
Predictors	*Estimates*	*CI*	*p*
(Intercept)	0.09	-0.31 – 0.49	0.656
Seeding.Strategy [Betweenness]	-0.03	-0.09 – 0.02	0.231
Seeding.Strategy [Complex]	0.09	0.04 – 0.15	**0.002**
Seeding.Strategy [K-core]	0.01	-0.04 – 0.07	0.664
Seeding.Strategy [Degree]	-0.02	-0.07 – 0.04	0.569
Seeding.Strategy [Eigen]	-0.02	-0.07 – 0.04	0.487
Seeding.Strategy [Percolation]	0.02	-0.04 – 0.07	0.521
nrooms	0.02	-0.01 – 0.05	0.207
nbeds	-0.02	-0.05 – 0.02	0.294
electricity	0.04	0.00 – 0.07	**0.038**
latrine	0.00	-0.03 – 0.03	0.859
nroomscapita	-0.08	-0.19 – 0.04	0.205
nbedscapita	0.02	-0.16 – 0.20	0.820
trimester	0.00	-0.04 – 0.05	0.837
Village Fixed Effects	✓	✓ ✓	✓
Observations	279		
R^2 / R^2 adjusted	0.530 / 0.424		

Supplementary Table 3. OLS model using each centrality measure to predict the fraction of each seed household's network neighborhood that adopted the BSS program (using all households as possible seeds), while controlling for all socioeconomic variables included in Banerjee et al.'s (2013) survey, with additional fixed effects at the village-level. The results are robust to varying the seeding strategy used as the referent strategy for the intercept.

Supplementary Table 3 replicates the model in Supplementary Table 2, while examining the capacity for each centrality measure to predict the fraction of seed household's neighborhood that adopted the BSS program, when using any potential adopting household as a seed (3). The intercept identifies the expectation when randomly identifying seed households. The results are

robust to varying the seeding strategy used as the referent strategy for the intercept. We see that, even when subject to all of the above controls, seeding with complex centrality is associated with a highly significant increase in the fraction of adopters relative to randomly selected households (p<0.01, $\beta_{complex}$ = 0.09¸CI=[0.03,0.14]). This effect still holds when clustering standard errors at the village level (p<0.05, $\beta_{complex}$ = 0.09). No other seeding strategies were identified as inducing a significance increase in the rate of adoption, relative to randomly selected seed households. Thus, again, we see that complex centrality significantly improves the capacity to identify influential households in the spread of the BSS program, beyond extant and state-of-the-art centrality measures. Overall, the above model accounts for 53% of the variance in the ability for households to trigger adoption of the BSS program among their network neighbors.

Supplementary References

1. Centola, D. & Macy, M. Complex Contagions and the Weakness of Long Ties. American Journal of Sociology 113, 702–734 (2007).
2. Jeon, K. C. & Goodson, P. US adolescents' friendship networks and health risk behaviors: a systematic review of studies using social network analysis and Add Health data. PeerJ 3, (2015).
3. Banerjee, A., Chandrasekhar, A., Duflo, E., & M. Jackson. The Diffusion of Microfinance. *Science* **341** (6144), 1236498 (2013).
4. Youm, Y., B. Lee, & Kim, J. A Measure of Centrality in Cyclic Diffusion Processes: Walk-Betweenness. *PLoS ONE* **16**(1), e0245476 (2021).
5. Freeman, L. C. A Set of Measures of Centrality Based on Betweenness. Sociometry 40, 35–41 (1977).
6. Newman, M. Networks: An Introduction. (Oxford University Press, 2010).
7. Bavelas, A. Communication Patterns in Task-Oriented Groups. Acoustical Society of America Journal 22, 725 (1950).
8. Mones, E., Vicsek, L. & Vicsek, T. Hierarchy Measure for Complex Networks. PLoS One 7, e33799 (2012).
9. Morone, F., & Makse, H. Influence Maximization in Complex Networks through Optimal Percolation. *Nature* **524** (7563), 65–68 (2015).
10. Carmi, S., Havlin, S., Kirkpatrick, S., Shavitt, Y., & Shir, E. A Model of Internet Topology Using K-Shell Decomposition. *Proceedings of the National Academy of Sciences* **104** (27), 11150–54 (2007).

11. Kempe, D., Kleinberg, J., & Tardos, É. Maximizing the Spread of Influence through a Social Network. *Theory of Computing* **11**, 105–147 (2003).
12. Holme, P. & Kim, B. J. Growing scale-free networks with tunable clustering. Physical Review E 65, 026107 (2002).
13. Barabási, A., & Albert, R. Emergence of Scaling in Random Networks. *Science* **286** (5439), 509–12 (1999).
14. Centola, D. *How Behavior Spreads*. (Princeton University Press, 2018).
15. Guilbeault, D., Becker, J., & Centola, D. Social learning and partisan bias in the interpretation of climate trends. *Proc. Natl. Acad. Sci. USA* **115**, 9714–9719 (2018).
